# BgTEP: An Antiprotease Involved in Innate Immune Sensing in *Biomphalaria glabrata*

**DOI:** 10.3389/fimmu.2018.01206

**Published:** 2018-05-29

**Authors:** Anaïs Portet, Richard Galinier, Silvain Pinaud, Julien Portela, Fanny Nowacki, Benjamin Gourbal, David Duval

**Affiliations:** Université de Perpignan Via Domitia, Interactions Hôtes Pathogènes Environnements UMR 5244, CNRS, IFREMER, Université de Montpellier, Perpignan, France

**Keywords:** thioester-containing protein, complement-like protein, *Biomphalaria glabrata*, interaction host/pathogens, innate immunity

## Abstract

Insect thioester-containing protein (iTEP) is the most recently defined group among the thioester-containing protein (TEP) superfamily. TEPs are key components of the immune system, and iTEPs from flies and mosquitoes were shown to be major immune weapons. Initially characterized from insects, TEP genes homologous to iTEP were further described from several other invertebrates including arthropods, cniderians, and mollusks albeit with few functional characterizations. In the freshwater snail *Biomphalaria glabrata*, a vector of the schistosomiasis disease, the presence of a TEP protein (BgTEP) was previously described in a well-defined immune complex involving snail lectins (fibrinogen-related proteins) and schistosome parasite mucins (SmPoMuc). To investigate the potential role of BgTEP in the immune response of the snail, we first characterized its genomic organization and its predicted protein structure. A phylogenetic analysis clustered BgTEP in a well-conserved subgroup of mollusk TEP. We then investigated the BgTEP expression profile in different snail tissues and followed immune challenges using different kinds of intruders during infection kinetics. Results revealed that BgTEP is particularly expressed in hemocytes, the immune-specialized cells in invertebrates, and is secreted into the hemolymph. Transcriptomic results further evidenced an intruder-dependent differential expression pattern of BgTEP, while interactome experiments showed that BgTEP is capable of binding to the surface of different microbes and parasite either in its full length form or in processed forms. An immunolocalization approach during snail infection by the *Schistosoma mansoni* parasite revealed that BgTEP is solely expressed by a subtype of hemocytes, the blast-like cells. This hemocyte subtype is present in the hemocytic capsule surrounding the parasite, suggesting a potential role in the parasite clearance by encapsulation. Through this work, we report the first characterization of a snail TEP. Our study also reveals that BgTEP may display an unexpected functional dual role. In addition to its previously characterized anti-protease activity, we demonstrate that BgTEP can bind to the intruder surface membrane, which supports a likely opsonin role.

## Introduction

Thioester-containing proteins (TEPs) are large secreted glycoproteins characterized by the presence of a unique intrachain β-cysteinyl-γ-glutamyl thioester bond (1). In native TEPs, this bond is unreactive, but following proteolytic activation, change in temperature, or aqueous conditions, the thioester bond becomes reactive and can bind closely accessible hydroxyl or amine groups that are present at the surface of many biological entities including pathogens (2).

The canonical intrachain thioester bond (GCGEQ) was originally described in the human alpha-2-macroglobulin (A_2_M), protease inhibitor, and C3, a central component of the complement system (3). Since then, members of the TEP superfamily have been identified and characterized in numerous distant phyla from Ecdysozoans and Lophotrochozoans to Deuterostomes. The TEP superfamily is divided into two subfamilies displaying distinct functions, complement factors, and A_2_M (2), supported by the presence of anaphylatoxin (ANA) and C-terminal C345C domains only in members of the complement factor subfamily. The complement factors subfamily contains human C3, C4, and C5 proteins, and their orthologs. The A_2_M subfamily comprises A_2_M, pregnancy zone protein-like (PZP), complement C3 and PZP A_2_M domain-containing 8 (CPAMD8), and cell surface glycoprotein CD109. In addition, two other classes of TEP were recently discovered in insects: the insect thioester-containing protein (iTEP) (4) and the macroglobulin complement-related (Mcr) (5), which constitute a third subfamily inside TEP superfamily, based on the phylogenetic analysis (2).

Thioester-containing proteins are key components of innate immunity: complement factors are deposited on the pathogen surface, enhance phagocytosis (opsonization), can recruit phagocytic cells at sites of infection (chemotaxis), and are capable to mediate pathogen lysis (6); while A_2_Ms are pan-protease inhibitors that sequester pathogen proteases, inhibiting their activity, and promoting their clearance (1, 7).

Thioester-containing proteins seem to appear early in animal evolution: members of this family are present in a wide variety of invertebrate species: in arthropods (8–12), crustaceans (13, 14), cnidarians (15), and mollusks (16, 17).

In invertebrates, TEPs have primarily been studied in *Anopheles gambiae* and *Drosophila melanogaster* (18, 19). The *A. gambiae* genome encodes 19 homologs of invertebrate TEP (iTEP) (AgTEP1–19), of which AgTEP1 is the best functionally characterized (19) and structurally the only crystallized TEP from invertebrates (20). AgTEP1 is reported to play an opsonin role in the phagocytosis of Gram-negative and Gram-positive bacteria (8, 21, 22). AgTEP1 also has the capacity to bind to the *Plasmodium* parasite surface to promote its melanization (19), and thus plays an essential role in decreasing the *Plasmodium* ookinete load in a mosquito’s gut (23). The *D. melanogaster* genome encodes six homologs of iTEP (DmTEP1–6) (9). Except DmTEP5, DmTEPs are expressed in immune tissues, i.e., hemocytes or fat body, and are upregulated after immune challenges with bacteria or yeasts (24–26). DmTEP2, DmTEP3, and DmTEP6 bind to Gram-negative bacteria, Gram-positive bacteria, and fungi, respectively, and play an opsonin role to promote the phagocytosis (5, 25). Furthermore, a mutant fly line lacking the four immune-inducible TEPs (TEP1–4) showed lower survival ability following Gram-positive bacteria, fungi, or parasitoid wasp immune challenges. This mutant fly line also presented a reduced toll pathway activation upon microbial infection, leading to a reduced antimicrobial peptide gene expression and thus a less efficient phagocytic response (27). Interestingly, another closely related protein Mcr has been identified for its ability to bind the cell surface of *Candida albicans* to promote its phagocytosis (5). This protein, characterized by the lack of the critical cysteine residue within the atypical thioester site (ESGEQN), is not processed during the interaction suggesting that the full-length protein is the active recognition form (5). Besides its role in immunity, Mcr is involved in autophagy regulation *via* the Draper immune receptor (28) and in septate junction formation (29, 30). More recently, a TEP has been identified in the shrimp *Litopenaeus vannamei* (LvTEP1), and its protective role against both Gram-positive and Gram-negative bacteria and viruses was highlighted by a knockdown approach (14). Also, the potential immune role of the *Chlamys farreri* TEP (CfTEP) has been suggested as CfTEP transcripts expression is induced following bacterial challenges, while CfTEP protein undergoes an apparent cleavage in a similar manner as the vertebrate’s C3 complement (31).

Here, we report the potential immune role of TEP protein from the freshwater snail *Biomphalaria glabrata* (BgTEP). In the last decade, *B. glabrata* has attracted attention due to its medical and epidemiological importance as a vector for schistosomiasis disease (32). Authors have invested considerable effort to investigate the molecular interactions and compatibility (susceptibility/resistance status) between *B. glabrata* and its parasite *Schistosoma mansoni* (33, 34) to help in the discovery of new ways to prevent and/or control schistosomiasis disease in the field (35). Co-immunoprecipitation experiments, using the previously characterized *S. mansoni* polymorphic mucins (SmPoMucs) as bait (36–38), enabled us to identify putative SmPoMuc-snail interacting immune receptors. An immune complex that associates three partners has been characterized: (1) SmPoMucs parasite molecules, (2) fibrinogen-related proteins (FREP), which is a highly diversified lectin family from snail hemolymph secreted by hemocytes and considered as pathogen-recognition receptors (39–41), and (3) a third partner, the newly identified *B. glabrata* TEP named BgTEP (42). The BgTEP is characterized by a stretch of cysteine residues at the C-terminal part and a highly conserved thioester motif (GCGEQ) of TEP family (42). Interestingly, BgTEP was firstly characterized as an alpha macroglobulin proteinase inhibitor by Bender and Bayne (43), who identified the very first N-teminal amino acids by Edman degradation. They demonstrated that BgTEP is able to inhibit in a methylamine-dependent manner, the activity of cysteine proteinases produced by *S. mansoni* miracidium and sporocysts.

In this study, we show that structural modeling prediction displays a strong similarity between BgTEP and AgTEP1, while the expression in different tissues reveals a wide distribution, showing a high abundance in snail hemocytes (circulating immune cells). Moreover, BgTEP displays a capacity to interact with diverse intruders, followed by a biochemical processing of the protein. Among hemocyte cells, BgTEP is secreted by a range of blast-like cells which are not phagocytic cells, but which are involved in the formation of the capsule surrounding the parasite. All of these results suggest that BgTEP may play a role in innate immune response against pathogens by assuming an opsonin function as evidenced in arthropods.

## Materials and Methods

### Ethical Statements

Our laboratory holds permit # A66040 for experiments on animals, which was obtained from the French Ministry of Agriculture and Fisheries and the French Ministry of National Education, Research, and Technology. The housing, breeding, and care of the utilized animals followed the ethical requirements of our country. The experimenter possesses an official certificate for animal experimentation from both of the above-listed French ministries (Decree # 87–848, October 19, 1987). The various protocols used in this study have been approved by the French veterinary agency of the DRAAF Languedoc-Roussillon (Direction Régionale de l’Alimentation, de l’Agriculture et de la Forêt), Montpellier, France (authorization # 007083).

### Biological Materials

The albino Brazilian strain snails (Recife, Brazil) were exposed to several microbes, Gram-negative bacteria culture of *Escherichia coli*, Gram-positive bacteria culture of *Micrococcus luteus* and yeast culture of *Saccharomyces cerevisiae*. Snails were also exposed to one Guadeloupean parasite strain of *S. mansoni* (le Lamentin, Guadeloupe).

This last interaction was chosen for its incompatibility (44), which means, the snail immune response is efficient and an encapsulation around the parasite is observed.

### Antibody Production

We used two types of antibody. The first is a purified polyclonal anti-BgTEP antibody produced in rabbits immunized with a synthetic peptide corresponding to the region 1238–1252 of BgTEP (the peptide sequence used is: H2N—SSY GSK SFR PDT NIT C—CONH2). IgG fraction was purified on an affinity column raised against the immunogenic peptide and antibody specificity was tested by Western blot. Production and purification of this antibody called anti-BgTEP-PEP was done according to standard procedures (Eurogentec, France) previously described in Ref. (42) and resuspended in PBS buffer (2.8 mg/mL). A second antibody called anti-BgTEP-RP was raised against a truncated recombinant protein which corresponds to the C-terminal part of BgTEP (amino acids 1036 to 1445). This truncated protein was histidine tagged at the N-terminus part by cloning the partial cDNA into the expression vector pET200/D-TOPO (Invitrogen). Recombinant protein production was performed with Bl21 (DE3) *E. coli* strain, and the purification was done by IMAC using a Ni-NTA column under native conditions as recommended by the manufacturer (Invitrogen) and as previously described in Ref. (42). The purified His6-tagged recombinant protein was then injected into chicken eggs to produce the polyclonal anti-BgTEP-RP antibody by the Agrobio Company (France). IgY fraction was purified by precipitation according to Agrobio Company procedure and resuspended in PBS buffer to a final concentration of 10.5 mg/mL.

### Genomic, Proteomic, and Structural Organization of BgTEP

The cDNA sequence of BgTEP, which has been previously characterized (42) (accession number HM003907), was BLASTed against VectorBase to characterize the exon–intron structure (Table S1 and Figure S1 in Supplementary Material). The 5′UTR and 3′UTR ends were determined using data of the Brazilian *B. glabrata* transcriptome (45). Prediction of the BgTEP three-dimensional (3D) structure and alignment with AgTEP1 (2PN5 published in 2015) (20) were performed using the I-Tasser and TM-align servers (46, 47). The 3D structure was obtained by multiple threading using the I-Tasser server (available online), which combines two protein structure prediction methods: threading and *ab initio* prediction (48). Structural similarities between the functional domain of AgTEP1 and BgTEP were determined by calculating a TM-score. A TM-score greater than 0.5 reveals significant alignment, whereas a TM-score less than 0.17 indicates a random similarity.

### Phylogenetic Organization of TEP Family Members

Homologous sequences were identified using BLAST searches against the GenBank non redundant database (Bethesda, MD, USA). For phylogenetic analyses, multiple protein sequence alignments were performed with Clustal W using the BLOSUM62 substitution matrix model. The neighbor-joining method (Poisson substitution model; uniform substitution rate; gaps/missing data treatment: pairwise deletion) was used to generate the phylogenetic tree, in order to cluster the different protein families, and to determine BgTEP position. Neighbor-joining method was chosen as full-length protein sequences of varying size and harboring different conserved domains were used to construct the tree. A bootstrap analysis of 2,000 replications was carried out to assess the robustness of the tree branches. A total of 125 full-length sequences from TEP superfamily proteins (Table S2 in Supplementary Material) were chosen to construct the tree based on the best BLAST matches against the NCBI database. The phylogenetic analysis was performed using MEGA 5 software (49). Besides, the search for a C-terminal transmembrane domain was performed to distinct CD109 from iTEP proteins, using the online transmembrane topology and signal peptide (SP) predictor Phobius from the Stockholm bioinformatic center (http://phobius.sbc.su.se/). The result of this search was used to classify invertebrate CD109-like proteins and iTEP in different subgroups in line with the phylogenetic tree obtained.

### BgTEP Interactome and Immunoblotting Approaches

#### *B. glabrata* Plasma Preparation

The hemolymph was extracted from the head-foot according to previously described procedures (50). After recovering the hemolymph, a first centrifugation for 5 min at 5,000 *g* at 4°C was performed to eliminate hemocytes then, an ultracentrifugation for 2.5 h at 40,000 *g* at 4°C leads to hemoglobin elimination. The ultracentrifuged plasma was only used for the study of the BgTEP profile in naive snail and to test the specificity of the different antibodies.

#### Interactome Samples Preparation

2 mL of fresh hemolymph was recovered from naive snails and centrifuged for 5 min at 5,000 *g* to eliminate hemocytes. In parallel, live microbe samples were recovered, centrifuged at 5,000 *g* for 5 min, and washed with Chernin’s balanced salt solution (CBSS) (NaCl 2.8 g/L, KCl 0.15 g/L, Na_2_HPO_4_ 0.07 g/L, MgSO_4_⋅7H_2_O 0.45 g/L, CaCl_2_⋅2H_2_O 0.53 g/L, NaHCO_3_ 0.05 g/L, pH 7.4) and kept at the bottom of the tube before use. Yeast and bacteria culture and preparation for interactome assays were performed as previously described (51). Depending on the intruder, samples were recovered from 500 µL of bacteria culture of *E. coli* and *M. luteus* (1.2 × 10^7^/mL), 500 µL of yeast culture of *S. cerevisiae* (7.5 × 10^5^/mL), and 500 miracidia (snail infective stage of *S. mansoni*) or 500 primary sporocysts (first snail-stage of *S. mansoni*). Two interactome conditions were then tested for each adopted intruder: intruders exposed either to (i) the host cell-free hemolymph fraction or to (ii) CBSS that mimics the internal host environment. Samples were incubated at 26°C (temperature of snail environment) for 30 min, to observe a rapid interaction between the BgTEP and the intruders, or 3 h to observe potential processing of BgTEP. Intruder samples incubated with hemolymph or CBSS buffer were then washed with CBSS as detailed above and pellet intruders were extracted using Laemmli SDS-PAGE buffer (Bio-Rad). The biological material was used to study the BgTEP profile after interaction between the cell-free hemolymph and intruders.

#### Methylamine Treatment

Fresh hemolymph was recovered from naive snails as described above. After hemocytes removing, hemolymph was pre-incubated with 2 mM methylamine (pH 6.4) for 2 h at 26°C using a rotating incubator. Then, 500 *S. mansoni* primary sporocysts were either incubated with 1 mL of CBSS buffer, or 1 mL of cell-free hemolymph, or 1 mL of cell-free hemolymph pre-incubated with 2 mM methylamine, for 30 min at 26°C under agitation for interaction. *S. mansoni* primary sporocysts were further washed with CBSS and proteins were extracted as described above. The presence or absence of BgTEP associated with the pathogen was revealed by Western blot.

#### Western Blot

8 µL of snail ultracentrifuged plasma or 10 µL of interactome samples was run on a 7.5% SDS-polyacrylamide precast gel (Mini Protean TGX Precast Gel Bio-Rad) for bacteria and yeast samples and 12% SDS-polyacrylamide precast gel (Mini Protean TGX Precast Gel Bio-Rad) for parasite samples and transferred onto a 0.2 µm PVDF membrane with Trans-Blot Turbo Transfer Pack (Bio-Rad). After saturation during 1 h at 37°C in TBSTM [1× TBS (500 mM Tris–HCl, 1.5 M NaCl, pH 7.5), 0.05% Tween-20, 5% non-fat milk], the protein blots were incubated for 1.5 h at room temperature in TBSTM, with a 1:200 dilution of purified anti-BgTEP-PEP antibody for the Western blot on ultracentrifuged naive snail plasma and a 1:2,000 dilution of the polyclonal anti-BgTEP-RP antibody for the interactome experiments. The blots were washed three times with TBST, and further incubated with 1:4,000 dilution of the commercial horseradish peroxidase-conjugated goat anti-rabbit IgG antibody (catalog number 32460, Thermo Fisher) for the Western blot on naive snail plasma, and 1:4,000 dilution of the commercial horseradish peroxidase-conjugated goat anti-chicken IgY antibody (catalog number 6100-05, SouthernBiotech) for the interactome experiments, in TBSTM for 70 min at room temperature. Blots were finally washed three times with TBST, and then revealed in the presence of an enhanced chemiluminescent substrate (SuperSignal West Pico Chemiluminescent Substrate, Thermo Fisher). These experiments were performed at least three times independently, and one representative blot is shown.

### BgTEP Immunolocalization in Hemocyte Populations

The hemolymph was extracted from the head-foot according to previously described procedures (50). The hemocytes were plated for 1 h on polystyrene chamber slides. Hemocytes were fixed using 4% paraformaldehyde for 10 min, followed by a cell permeabilization step with 0.01% Triton X-100 and 3% BSA for 20 min. Cells were incubated with a 1:100 dilution of anti-BgTEP-PEP for 1 h followed by a 1:1,000 dilution of the manufactured fluorescent goat anti-rabbit IgG antibody (Thermo Fisher, Alexa Fluor 594) for 50 min. After the BgTEP labeling, the actin was labeled with (Thermo Fisher, Alexa Fluor 488) Phalloidin (Thermo Fisher) for 20 min and the cell nucleus with Dapi (Biotum) for 30 s. Observations were performed by confocal microscopy using a Zeiss LSM 700 microscope at 405, 488, or 555 nm. These experiments were performed at least 10 times independently with the anti-BgTEP-PEP antibody. Negative controls were done to confirm the specificity of the anti-BgTEP-PEP antibody, i.e., using either secondary antibody alone or preincubating the anti-BgTEP-PEP antibody with the immunogenic peptide. No cell labeling was observed in the negative control condition.

### Phagocytosis Assay

Approximately 1 × 10^6^ particles (5 µL) of zymosan A conjugated with an Alexa Fluor 488 (Invitrogen, BioParticles Z23373) in CBSS were injected into snails. The hemolymph was recovered 3 h post-injection. The hemocytes were plated on chamber-slides and prepared as described above. Two types of labeling were performed. Either, an alexa fluor 594 phalloidin was used to label actin of all immune cells or the anti-BgTEP-PEP antibody detected using Alexa Fluor^®^ 594 conjugated goat anti-Rabbit IgG secondary antibody (Thermo Fisher, catalog number A-11012) to reveal BgTEP-positive cells. The internalized green bioparticles in different hemocytes were monitored by several focal observations using the Zeiss LSM 700 confocal microscope. These experiments were performed at least three times independently.

### Fluorescent Staining and Flow Cytometry Method

The hemolymph was extracted as described above. Three biological replicates (pools of 15 snails per replicate) were performed. Hemocytes were fixed in suspension using 4% paraformaldehyde for 10 min, followed by a cell permeabilization step with 0.01% Triton X-100 and 3% BSA for 20 min. Cells were incubated with a 1:100 dilution of anti-BgTEP-PEP for 1 h followed by a 1:1,000 dilution of the manufactured fluorescent goat anti-rabbit IgG antibody (Thermo Fisher Scientific—Alexa Fluor 594) for 50 min. Hemocyte population was profiled along using Side Scatter Channel and Forward Scatter Channel (FSC) to estimate cell granularity and cell size, respectively. The PE channel was used to detect light emitted from Alexa Fluor 594 dye conjugated to the secondary antibody. The flow cytometry was performed using a FACSCanto from BD Biosciences (RIO Imaging Platform, Montpellier, France). The threshold was determined according to the PE channel and the FSC parameter. Note that the largest cells tend to slightly autofluorescence. For each sample, about 10,000 events were counted. The results were analyzed using the FlowJo V 10.0.8 software.

### BgTEP Expression Analysis

#### RNA Extraction and Quantitative RT-PCR Protocol

Snail total RNA was extracted with TRIzol reagent (Sigma Life Science) according to the manufacturer’s instructions and subsequently reverse transcribed to first strand cDNA using Maxima H Minus First Strand cDNA Synthesis Kit with dsDNase (Thermo Scientific) according to the manufacturer’s instructions.

Real-time RT-PCR analyses were performed using the LightCycler 480 System (Roche) in a 10 µL volume comprising 2 µL of cDNA diluted to 1:200 with ultrapure-water, 5 µL of No Rox SYBR Master Mix blue dTTP (Takyon), 1 µL of ultrapure-water, and 10 µM of each primer. The primers used for the RT-QPCR are TEP-R: ACCATTAGATCCACTGGAAGATA TEP-F: CTGACTTACCCTCGCTC for BgTEP, and S19-R: CCTGTATTTGCATCCTGTT S19-F: TTCTGTTGCTCGCCAC for S19 ribosomal protein gene used as housekeeping gene. The two primer couples have been tested to determine the exponential and efficiency of the PCR reaction. The cycling program is as follows: denaturation step at 95°C for 2 min, 40 cycles of amplification (denaturation at 95°C for 10 s, annealing at 58°C for 20 s, and elongation at 72°C for 30 s), with a final elongation step at 72°C for 5 min. QPCR was ended by a melting curve step from 65 to 97°C with a heating rate of 0.11°C/s and continuous fluorescence measurement. For each reaction, the cycle threshold (*C*_t_) was determined using the second derivative method of the LightCycler 480 Software release 1.5 (Roche). PCR experiments were performed in triplicate (technical replicates) from four biological replicates. The mean value of *C*_t_ was calculated. Corrected melting curves were checked using the *T*_m_-calling method of the LightCycler 480 Software release 1.5.

#### Tissue Recovery

Tissues were collected from 9 to 10 snails under binocular microscope dissection. Albumen gland, head-foot, hepatopancreas, and ovotestis were recovered. For hemocytes recovery, the hemolymph of 50 snails was collected, and cells were recovered after centrifugation at 10,000 *g* for 10 min. The relative expression of BgTEP was calculated with the E-method which accounts the efficiency of both couple of primers. Results were normalized with respect to a housekeeping gene the S19 ribosomal protein gene, as previously described (52). Statistical analysis was done by a one-way ANOVA test followed by a *post hoc* Games–Howell pairwise comparison test.

#### Infection by Multiple Intruders of Whole Snails

Contact with Gram-positive and Gram-negative bacteria and yeast were established according to previously described procedures (53). Briefly, snails were bathed with 10^8^/mL of microbes for 1 h, then snails were washed. For the parasite infection, each snail was exposed for 6 h to 10 miracidia in 5 mL of pond water. For each intruder stimulation, 36 snails were used: 4 independent replicates using a pool of 3 snails were performed at 3 time points (6, 12, and 24 h after stimulation). As control condition, four replicates using a pool of three snails were recovered for the evaluation of the basal expression of BgTEP. The relative expression of BgTEP was normalized with respect to a housekeeping gene the S19 ribosomal protein gene, and BgTEP expression was compared with non-exposed snails. The significant difference in BgTEP expression was evaluated based on Δ*C*_t_ values, according to a Kruskal–Wallis test and followed by a Dunn’s *post hoc* test.

PCR experiments were performed in triplicate (technical replicates) from four biological replicates. The mean value of *C*_t_ was calculated. Corrected melting curves were checked using the *T*_m_-calling method of the LightCycler 480 Software release 1.5.

### *In Situ* Histological Localization of BgTEP

#### Snail Infection

Snail were infected with the parasite strain as previously described, briefly each snail was exposed for 6 h to 10 miracidia in 5 mL of pond water.

#### Immunocytochemistry Procedures

Snails were fixed in Halmi’s fixative (4.5% mercuric chloride, 0.5% sodium chloride, 2% trichloroacetic acid, 20% formol, 4% acetic acid, and 10% picric acid-saturated aqueous solution). Embedding in paraffin and transverse histological sections (10 µm) were performed. The slides were stained using Anti-BgTEP-PEP antibody according to a previously developed protocol (36).

Slides re-hydration was performed in serial toluene, 95, 70, and 30% ethanol and finally PBS bathing (saline phosphate buffer: pH 7.4–7.5; 8.41 mM Na_2_HPO_4_; 1.65 mM Na_2_H_2_PO_4_; 45.34 mM NaCl; H_2_O milliQ q.s.p.). The slides were immersed in a permeabilizing PBS solution containing 0.5% Triton X-100 during 15 min. A saturation step was performed in PBS buffer containing 1% gelatin hydrolyzate (Bellon, France), 1% normal goat serum (NGS, Sigma), and 0.1% NaN_3_ (Sigma) during 1 h at room temperature. Slides were then successively incubated with the anti-BgTEP-PEP antibody dilution 1:100 for 1 h 30 min at room temperature and with an Alexa Fluor 594 anti-rabbit IgG (Thermo Fisher Scientific) diluted 1:1,000 for 1 h at room temperature. Slides were mounted in Vectashield and stored in dark at 4°C. The slide observation was carried out by epifluorescence and light microscopy using a Zeiss axioscope 2 microscope (Carl Zeiss AG) and a Leica DC350FX camera (Leica).

## Results

### Organization of BgTEP

**Figure 1:**
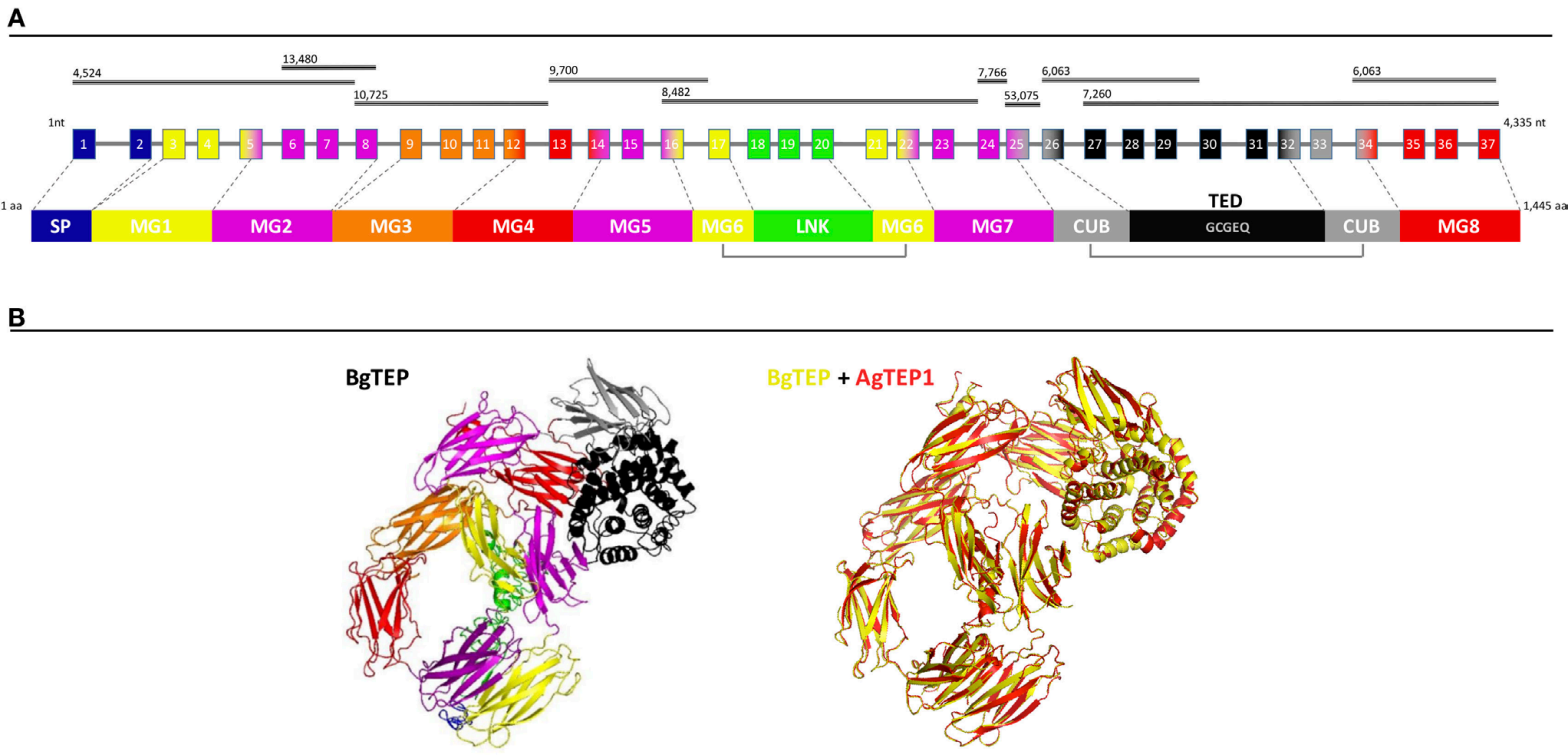
BgTEP organization. **(A)** Schematic representation of BgTEP sequence organization. The top black lines correspond to *Biomphalaria glabrata* genome scaffolds assigned with their corresponding scaffold numbers. The center part is a schematic of the exon/intron organization of BgTEP gene. The squares represent exons, the lines for introns, the number corresponds to the exon number, and finally their associated color corresponds to the different protein domain encoded that mapped on three-dimensional (3D) structure. The lower part corresponds to the schematic domain arrangement of BgTEP protein. The signal peptide (SP) domain is represented in blue followed by macroglobulin domain 1 (MG1) in yellow, MG2 in magenta, MG3 in orange, MG5 (magenta), MG6 (yellow), linker domain (LNK) (green), MG7 (magenta), CUB (gray), thioester domain (TED) with conserved motif (GCGEQ) in black, and MG8 in red. **(B)** Overview of 3D BgTEP structure predicted by I-Tasser software, using *Anopheles gambiae* TEP1 (AgTEP1) as reference. The colored domains of the BgTEP protein match the color of the corresponding encoding exon. Despite a low similarity at the amino-acid level (29%), a high conserved spatial conformation (TM-score = 0.98) was observed between BgTEP (yellow) and TEP1 of *A. gambiae* (red).

BgTEP exhibits all the characteristics of known iTEP family members, including a SP for secretion, several predicted N-glycosylation sites, the canonical thioester motif (GCGEQ), the complement component domain (pfam PF07678), the Alpha2 macroglobulin receptor binding domain (pfam PF07677), and numerous cysteins such as the six C-terminal ones which are a signature of iTEPs (42). At the genomic level, BgTEP is composed of 37 exons spreading on 10 scaffolds of the *B. glabrata* genome assembly (54) (Figure 1A). Exon–intron boundaries were conserved along the sequence (Table S1 in Supplementary Material). At the secondary structure level, BgTEP protein possesses an N-terminal alpha helix characteristic of SP for secreted proteins, like other iTEPs. As expected, BgTEP is composed of eight MG (macroglobulin) domains, a succession of several β-sheets related to the fibronectin type III (fnIII) domains, with insertions of a LNK domain nested into MG6 and a CUB domain (β-sheet domain), and of a thioester domain (TED) (α-helical thioester domain) between MG7 and MG8 (Figure 1A).

The BgTEP protein’s tertiary structure was also investigated with the structure prediction software I-Tasser (Figure 1B), using AgTEP1 protein from *A. gambiae* as reference structure. The score obtained for this prediction is highly significant (TM-score = 0.98), indicating the robustness of the prediction, as well as a structural alignment conservation with the AgTEP1 reference despite a low similarity at the amino-acid level (29%) (Figure 1B).

### Phylogenetic Analysis of BgTEP

**Figure 2:**
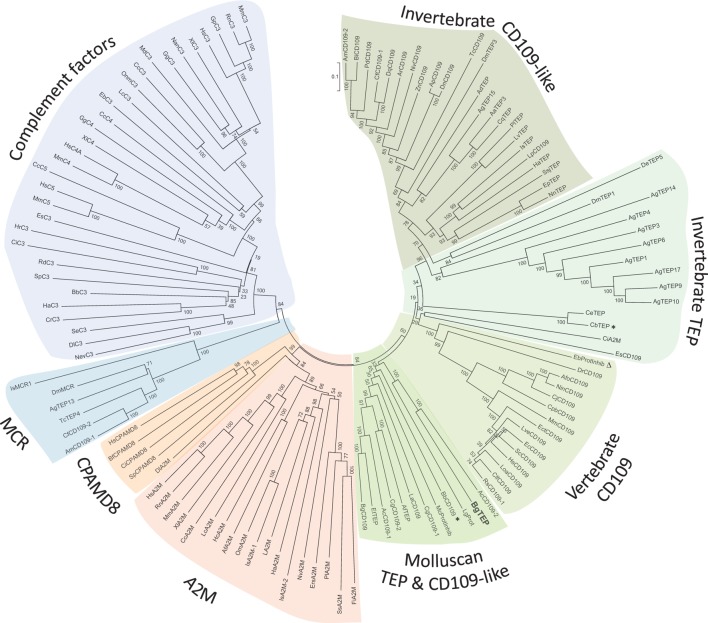
Phylogenetic tree of thioester-containing protein (TEP) superfamily. Phylogenetic analysis of the TEP superfamily full-length protein sequences from 125 members (see Table S2 in Supplementary Material). Complement factor groups are colored in blue shades, alpha-2 macroglobulin groups in red shades, and insect thioester-containing protein and CD109 groups in green shades. BgTEP is indicated in bold type. A bootstrap analysis of 2,000 replications was carried out on the tree inferred from the neighbor-joining method and the values are shown at each branch of the tree.

Since the first identification of BgTEP in 2010 (42), many new members of the TEP superfamily have been discovered, including several from the phylum *Mollusca*. In response to these recent discoveries, we conducted a new phylogenetic analysis to position BgTEP among the main TEP family members. To do this, we used 125 amino acid sequences of full-length TEP superfamily proteins (see Table S2 in Supplementary Material), including complement-like factors, macroglobulin complement-related proteins (Mcr), A_2_M, complement 3 and pregnancy zone protein-like A_2_M domain-containing 8 (CPAMD8), CD109 glycoproteins, and iTEP proteins, to construct a phylogenetic tree with the neighbor-joining method (Figure 2). Phylogenetic analysis confirms the localization of BgTEP within the group of iTEPs. This analysis segregates the proteins into three major groups: the complement component group which includes complement and complement-like factors as well as Mcr (Figure 2, blue shades), the A2M group comprising A2M and CPAMD8 (Figure 2, red shades), and the group formed by cell surface TEP (CD109) and iTEP (Figure 2, green shades). However, the analysis reveals several subgroups within the iTEP and CD109 group. Vertebrate and invertebrate CD109 proteins form two distant subgroups, while invertebrate TEP and invertebrate CD109 form two closely related subgroups. CD109-like proteins from invertebrate may be distinguished from iTEP proteins by the presence of a putative C-terminal transmembrane domain (13), as reported for the vertebrate GPI-anchored CD109 proteins (55). Moreover, BgTEP is included in an additional well-supported subgroup constituted by both CD109 and iTEP from mollusk species, reflecting a degree of similarity in the sequences of TEP proteins from the phylum Mollusca. The mix of CD109 and iTEP in the same subgroup is likely the result of automatic—and not manual—corrected annotation from Genbank without further characterization.

### BgTEP Expression in Snail Tissues

**Figure 3:**
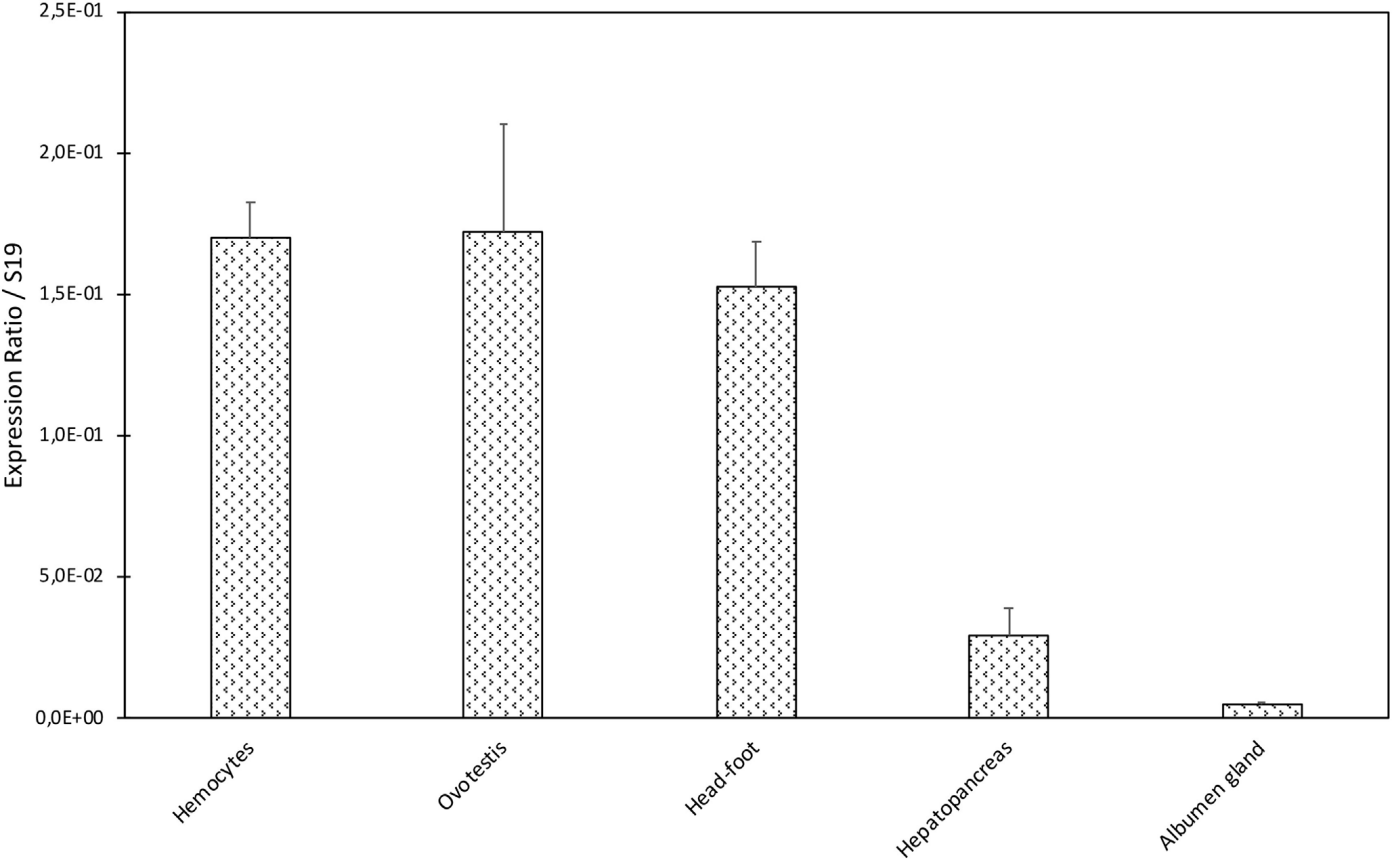
Tissue expression of BgTEP. Transcription profile of BgTEP in different tissues extracted from 9 to 10 snails. Five snail tissues are dissected: albumen gland, hepatopancreas, head-foot, and ovotestis. Hemocytes were collected from 50 snails. Quantitative RT-PCR was performed on total RNA. *C*_t_ values of the BgTEP transcript were normalized to the transcript level of the reference gene S19. Error bars represent the SD of the Δ*C*_t_ mean values obtained for each tissue. Asterisks indicate a significant expression difference between mentioned tissues.

A dissection of different organs (hemocytes, ovotestis, head-foot, hepatopancreas, and albumen gland) of *B. glabrata* was conducted to analyze tissue distribution of BgTEP by quantitative RT-PCR (Figure 3). BgTEP was expressed in all tissues examined with different levels; a high expression is observed in hemocytes, ovotestis, and head-foot, whereas a lower expression is observed in hepatopancreas and albumen gland (Figure 3).

**Figure 4:**
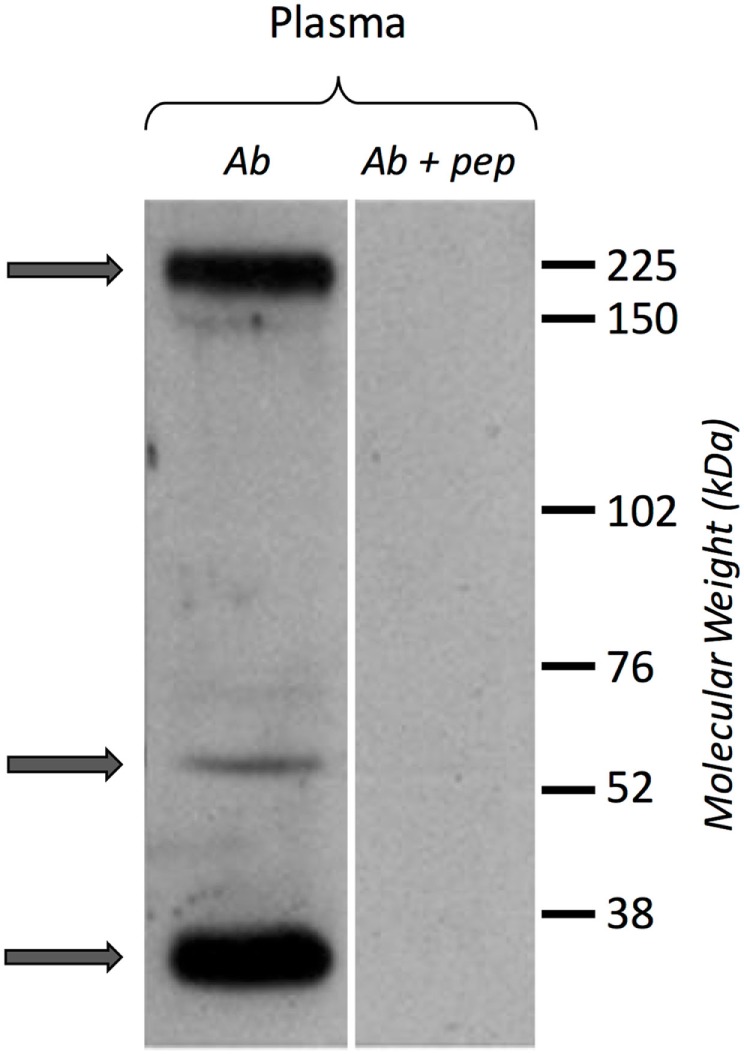
BgTEP secretion. Left panel: presence of BgTEP protein on hemolymphatic cell-free compartment was tested by Western blot using an affinity-purified polyclonal antibody (anti-BgTEP-PEP). Ultracentrifuged hemolymph was used to remove hemoglobin. Right panel: as negative control, anti-BgTEP antibody and BgTEP peptide (10-fold molar excess) pre-incubation was performed before incubation with the membrane (Ab + pep). Arrows correspond to the full-length and two processed forms of BgTEP protein present in hemolymph from naïve snails.

A Western blot experiment was performed on ultracentrifuged cell-free hemolymph from naïve snails with a polyclonal anti-BgTEP antibody, designed from a C-terminal peptide, and called anti-BgTEP-PEP. We detected three bands of BgTEP in naive plasma (Figure 4). The first band abundantly detected at approximately 200 kDa corresponds to the full-length BgTEP (Figure 4). Two lower bands (60 and about 30 kDa) were interpreted as BgTEP processed forms, resulting from a fine regulation of proteolysis by endogenous proteases observed also in mosquito plasma (Figure 4).

### BgTEP Expression After Immune Challenges

**Figure 5:**
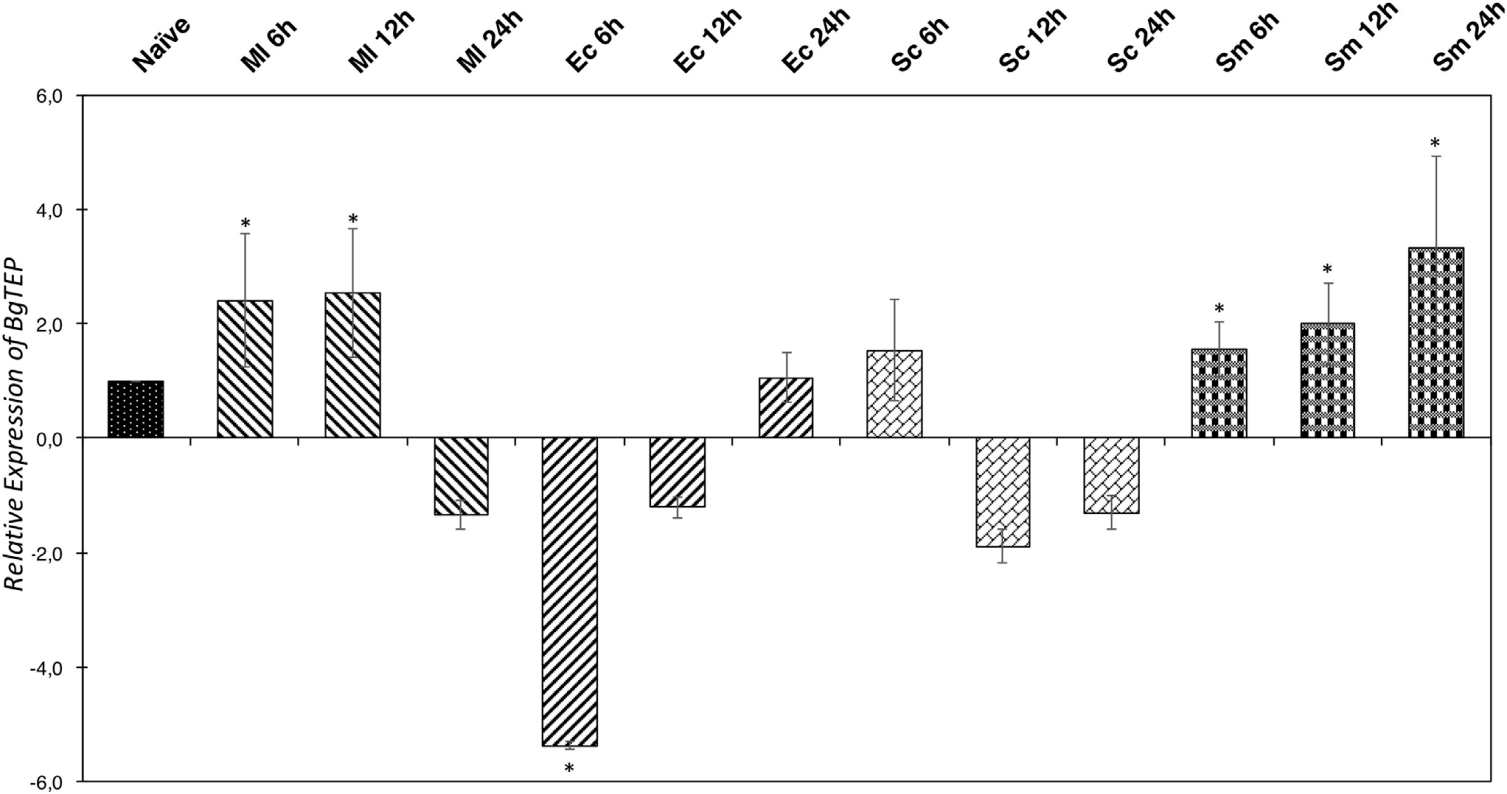
Immune-inducible transcription of BgTEP. Quantitative RT-PCR was performed from whole snails exposed to different immune challenges or unexposed. BgTEP expression was monitored at three time points (6, 12, and 24 h) post-challenge with *Micrococcus luteus* (decreasing hatching), *Escherichia coli* (increasing hatching), *Saccharomyces cerevisiae* (rectangle), and *Schistosoma mansoni* (square). BgTEP expression was normalized by S19 housekeeping gene expression for each experimental point and compared with the relative expression obtained in non-exposed snails. Error bars represent the SD of the relative quantification values obtained for each kinetic point. The significant difference in BgTEP expression was evaluated according to a Kruskal–Wallis test followed by a Dunn’s *post hoc* test. The asterisks indicate a significant difference between non-exposed and exposed snails.

In some invertebrate models, the iTEP has been suggested to play a role during immune infections, mostly assuming an opsonin function. To investigate this potential function in our *B. glabrata* model, we measured the relative expression ratio by RT-QPCR of BgTEP transcripts in response to different immune challenges (Figure 5). The expression varies greatly depending on intruder immune challenges but also during infection kinetics. Indeed, BgTEP expression is decreased after both *E. coli* and *S. cerevisiae* challenges, while it is upregulated after *M. luteus* and *S. mansoni* stimulations (Figure 5). When challenging with *E. coli*, the expression is rapidly lowered nearly fivefold and returns to basal expression level after 24 h. However, while following the *S. cerevisiae* challenge, BgTEP expression is only slightly decreased twofold from 12 to 24 h post-challenge. For the other challenges, we observed a 2-fold increase in expression from 6 to 12 h after *M. luteus* stimulation, while for the *S. mansoni* challenge, the BgTEP expression increases regularly from 6 to 24 h from 2- to 3.5-fold.

As the number of BgTEP transcripts is regulated following challenges with different intruder types, we wonder if this protein might be involved in pathogen recognition, like in other invertebrate models such as *Drosophila* or *Anopheles*, that play an opsonin role in immune recognition response.

### BgTEP Capacities to Link to Intruder Surface

**Figure 6:**
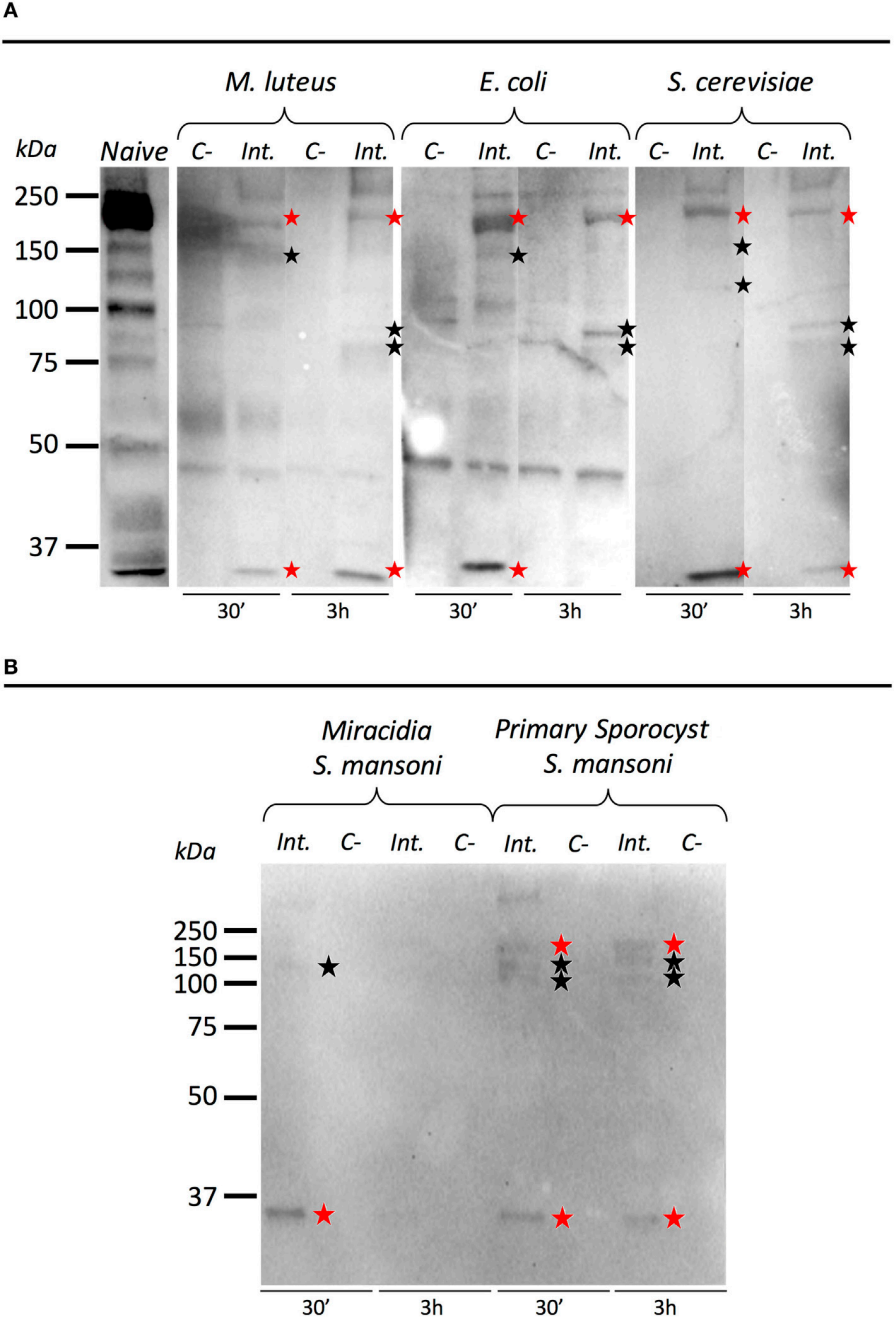
Immunoblotting analysis of BgTEP. Live intruders interaction with *Biomphalaria glabrata* cell-free hemolymph was monitored by Western blot using anti-BgTEP-RP antibody. Ultracentrifuged cell-free hemolymph from naïve snail was used as positive control for full-length and processed forms detection of BgTEP. For each interaction condition, negative control (C−) corresponds to the intruder alone, interactome (Int.) corresponds to the interaction between the intruder and *B. glabrata* cell-free hemolymph. Red stars correspond to putative full-length (200 kDa) and cleaved protein (about 30 kDa) observed in all interactions, and black stars indicate bands between 75 and 150 kDa corresponding to BgTEP alternative processed forms. **(A)** Comparison of BgTEP binding and time-dependent processing after 30 min and 3 h of interaction between cell-free hemolymph and *Micrococcus luteus, Escherichia coli*, and *Saccharomyces cerevisiae*. **(B)** Comparison of BgTEP binding between two developmental stages of *Schistosoma mansoni* (miracidia and primary sporocyst).

To investigate the ability of BgTEP to bind to the intruder surface during infections, we performed interactions at 26°C between *B. glabrata* cell-free hemolymph and *M. luteus, E. coli, S. cerevisiae*, or *S. mansoni* parasites (Figure 6). As a comparison, intruders alone were incubated at the same time in CBSS buffer (negative control) (Figure 6). The experiments were performed with whole live intruders allowing for two interaction times (30 min and 3 h) in order to detect the binding and the potential processing of BgTEP protein during binding kinetics. Once the interactions were complete, intruders were recovered by centrifugation, washed, and bound BgTEP was revealed by Western blot using the anti-BgTEP-RP antibody (Figure 6). The antibody raised against the C-terminal part of the BgTEP revealed several bands in positive control, three of which (200, 100, and 50 kDa) were more intense (Figure 6A, lane 1).

Interestingly, we demonstrate that the full length of BgTEP (band of 200 kDa) binds all bacteria, yeast (Figure 6A), and metazoan parasites (Figure 6B). Surprisingly, we also observed that a short processed form of TEP (about 30 kDa) present in naïve hemolymph can also bind to all intruders. Other minor bands were detected by the anti-BgTEP-RP antibody from 75 to 150 kDa with *E. coli, M. luteus*, and *S. cerevisiae* that differed between 30 min and 3 h interactions (Figure 6A).

We investigated the interaction between BgTEP and two different development stages of *S. mansoni* parasite; the miracidia, a free-living and swimming larva that infests the host snail, and the primary sporocyst (Sp1) which is the first intra-molluscal stage of the parasite after infestation (Figure 6B). For the interaction with *S. mansoni*, we demonstrate that the full length of BgTEP binds the parasite at the two development stages, but to a lower extent with miracidia stage (Figure 6B). We also observed that the short processed form of BgTEP (30 kDa) binds the parasite but is no longer detected with miracidia stage at the 3-h interaction (Figure 6B). Moreover, minor bands were detected from 100 to 150 kDa for the primary sporocyst stage all along the interaction, whereas only a weak band is observed for the miracidia stage (Figure 6B). This discrepancy between the two profiles suggests different BgTEP binding and processing abilities for the two parasite stages.

Moreover, we performed a 2 mM methylamine treatment of cell-free hemolymph in order to determine if the binding of full length and processed forms of BgTEP to *S. mansoni* primary sporocyst is dependent or not of the thioester site. Results (Figure S3 in Supplementary Material) showed that the binding of full-length BgTEP as well as of the processed form observed at 100 and 135 kDa to Sp1 is drastically affected by the used of methylamine, and so do depend to the thioester site.

To conclude, we evidence the binding of BgTEP to all of the tested intruders but also a differential BgTEP intruder-dependent processing that occurs during interaction. The disappearance or the presence of new forms detected on the intruder surface highly suggest that BgTEP likely has a complex modification of its structure with a possible fine proteolytic regulation required for opsonization or/and encapsulation.

### BgTEP Expression in Hemocyte Subpopulations

**Figure 7:**
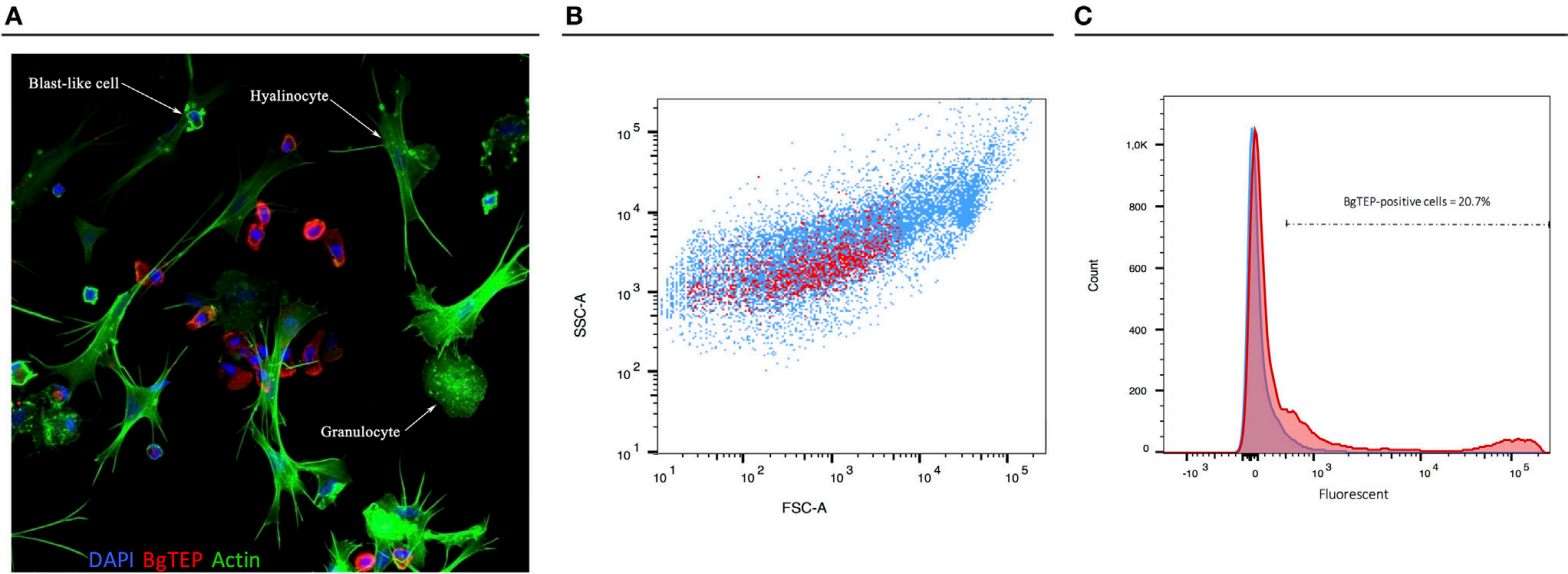
Cell-type expression of BgTEP. **(A)** Immunolocalization of BgTEP in hemocyte populations by confocal microscopy. Three major cell types are present in *Biomphalaria glabrata* hemolymph: the hyalinocytes with cytoplasmic projections, the granulocytes with granules into the cytoplasm, and the blast-like cells, the smallest cells (Figure S2 in Supplementary Material). Detection of intracellular BgTEP was performed by immunolocalization using the antibody anti-BgTEP-PEP. Alexa-488 phalloidin was used to visualize actin filaments (green) and DAPI for nuclear staining. The red labeling corresponds to BgTEP detection. **(B)** Flow cytometry profile of BgTEP-positive hemocytes in hemolymph. The total hemocytes are shown according to their size Forward Scatter Channel (FSC) and granularity Side Scatter Channel (SSC). The red dots correspond to BgTEP-positive hemocytes. **(C)** Flow cytometry quantification of BgTEP-positive cells present in hemolymph. Negative control was performed using the conjugated secondary antibody alone (blue). A fluorescent cutoff was determined to count BgTEP-positive cells.

Since BgTEP can bind different intruders and display an opsonin role in other models, we focused on immune activities at the cellular level. First, we performed an immune-labeling experiment upon plated hemocytes to investigate which subtype expresses BgTEP (Figure 7A). Immunolocalization shows that not all hemocytes produce and secrete BgTEP and its expression is only restricted to a subset of blast-like cells (Figure 7A). Moreover, to estimate the BgTEP-positive blast-like cell proportion in hemocytes, we performed a cell quantification by flow cytometry (Figure 7B). The BgTEP-positive cells correspond to about 20% of total hemocytes (Figure 7C). This result has been confirmed by microscopy cell counting. About 50% of blast-like cells, which represent approximately half of the entire population of hemocytes (Figure S2 in Supplementary Material), are BgTEP-positive cells.

### Potential Role of BgTEP-Positive Hemocytes in Phagocytosis and/or Encapsulation Processes

**Figure 8:**
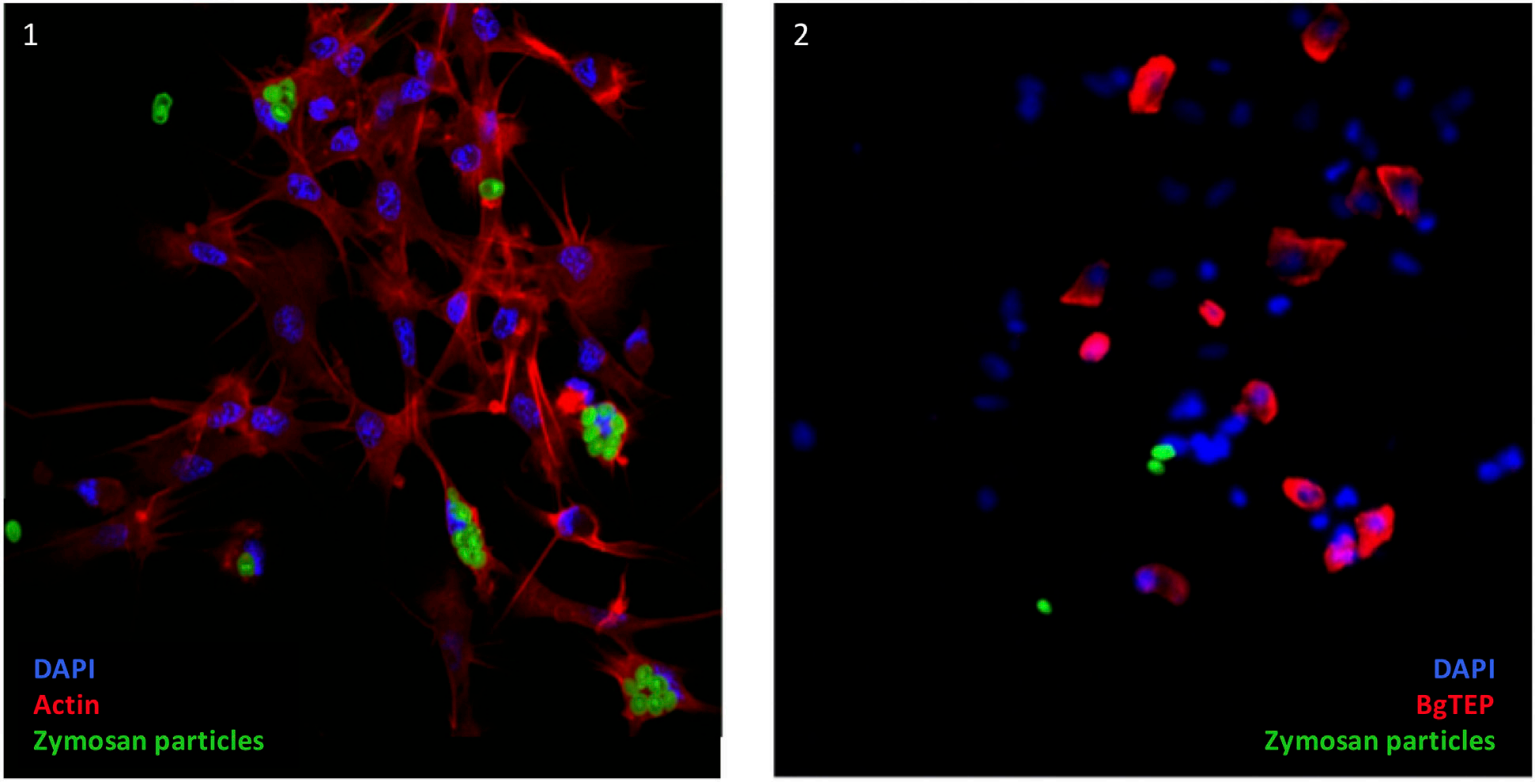
BgTEP-positive cells do not phagocyte. Phagocytosis of green fluorescent zymosan particles in hemocytes observed by confocal microscopy. On picture 1, Alexa-594 phalloidin was used to label actin of all hemocytes (red). Phagocytosis assay was monitored using green fluorescent yeasts. On picture 2, BgTEP-positive cells were detected by immunolocalization using the antibody anti-BgTEP-PEP and an Alexa Fluor 594 dye conjugated to the secondary antibody (red).

As some immune cells express BgTEP, we focused on its phagocytosis and encapsulation role. A phagocytosis experiment was performed using green fluorescent zymosan particles. These bioparticles were injected *in vivo* in snails, and after 3 h, the plasma was recovered to observe phagocytosis by confocal microscopy (Figure 8). Interestingly, zymosan phagocytosis was observed but only in immune cells that do not express BgTEP. Among immune cells, we did not observe phagocytosis in granulocytes and blast-like cells. The same approach was applied using *E. coli* and *S. aureus* bacteria, and no phagocytosis was observed in blast-like cells, for those that expressed the BgTEP and for those that did not (data not shown). In conclusion, hemocytes that express BgTEP are not directly involved in the microbe phagocytosis but must instead secret an opsonin factor capable of binding to the intruder surface and of facilitating its elimination in cooperation with other immune cell subtypes.

Finally, the role of BgTEP in parasite encapsulation was assessed through *in situ* immunocytochemistry localization. Following infection of *B. glabrata* by an incompatible *S. mansoni* parasite, the snail mounted a cellular immune response resulting in the encapsulation of the parasite by hemocytes. Histological sections of *S. mansoni*-infected snails were performed after 24 h post-exposure. Using the anti-BgTEP-PEP antibody, we observed a diffuse labeling around the parasite into the capsule (Figure S4 in Supplementary Material). This suggests that BgTEP gets in contact with the parasite, which is consistent with the *in vitro* binding demonstration of BgTEP to primary sporocyst surface (Figure 6B). We also observed numerous BgTEP-positive hemocytes recruiting to the site of encapsulation, as well as in the vicinity of the capsule and inside the capsule. A less intense labeling is further observed in head-foot cell wall, which is in line with the BgTEP expression measured by QRT-PCR in this tissue. These results support the hypothesis that BgTEP plays a sensing role, which could in turn promote the recruitment of other hemocyte subtypes required to mount an efficient phagocytosis or encapsulation immune response.

## Discussion

BgTEP characterization started 25 years ago with a first report of a proteinase inhibitory activity in the plasma of *B. glabrata* (56). In this study, we describe an alpha macroglobulin with trypsin inhibitory activity that is sensitive to methylamine treatment (43, 57). We succeeded in purifying the protein responsible for these activities and obtained the first 18 amino acids by Edman degradation sequencing. This sequence corresponded to the N-terminal part, excluding its SP of BgTEP, defined in 2010 as a third actor in immune complex between snail plasmatic lectin proteins, the FREP and parasite mucins, the SmPoMuc (42). A detailed biochemical characterization showed that this antiprotease is structured as a tetramer and undergoes a conformational change after protease cleavage leading to the activation of the thioester bond. The foreign protease is then sequestered and cannot react with bulky substrates (43).

The BgTEP protein sequence was characterized and displayed all the defining features of invertebrate TEP proteins with eight MG domains, nested insertion of a CUB domain, the canonical TED domain, several putative N-glycosylation sites, and the C-terminal signature composed of six cysteine residues (Figure 1).

Herein, we explore the molecular involvement of BgTEP in the innate immune response of the freshwater snail *B. glabrata* against a large panel of intruders. To elucidate the nature of BgTEP within the context of the TEP superfamily, we performed a phylogenetic analysis based on full-length amino acid sequences that segregated the three main groups: complement factors, A_2_M, and TEP/CD109 (Figure 2). BgTEP clearly clusters within the iTEP/CD109 group and does not cluster with the A_2_M group, despite the previous report of BgTEP proteinase inhibitory activity (43). Interestingly, a subgroup is formed with both iTEPs and CD109 from mollusks, which clusters more distantly from iTEP and CD109 proteins of all other phyla. This may be related to the primary sequence similarities between mollusk iTEPs or may be due to a functional specificity. Among this mollusk subgroup, many proteins are predicted from automatic genome annotations, which means that they were not previously characterized, with the exception of clam TEP (16) and bobtail squid CD109 (17). A unique sequence from this subgroup carries a predicted C-terminal transmembrane domain, while most of them were automatically annotated as CD109 proteins. A deeper characterization and re-annotation of each of these molecules deserves further investigation.

We then investigated the intron–exon structure of the BgTEP gene using the recently published *B. glabrata* genome (54). We identified 37 exons distributed on 10 different scaffolds from the genome assembly (BglaB1 assembly) (Figure 1). Such organization is consistent with the one of human TEP gene families like human CD109, A_2_M, and C3 complement factor genes that, respectively, encompass 33, 36, and 41 exons (58) and the TEP of the invertebrate *Chlamys farreri* with 40 exons. Moreover, the phylogenetic analysis of the TEP superfamily reflects a mollusk TEP group that is close to vertebrate TEP and CD109 suggests a lower evolutionary divergence than with other TEPs (59). Indeed, the BgTEP gene is considerably different from the AgTEP1 gene that is composed of only 11 exons (personal communication from VectorBase), thus suggesting a strikingly different evolutionary story between snail and mosquito TEPs. Despite these differences, it is not clear whether phylogenetic proximity and genomic organization are linked to TEP function as TEP activity is more conditioned by its quaternary structure than by its primary sequence (1). A structural protein prediction and alignment reveals a very close conformation between AgTEP1 and BgTEP. This result reflects a potential common function between those two complement-like components which otherwise display a low primary sequence similarity. AgTEP1, which is the most studied and the only crystallized invertebrate TEP, was shown to opsonize Gram-negative and Gram-positive bacteria and to promote their phagocytosis (8). AgTEP1 was also shown to target *Plasmodium* parasites for lysis through a hemocyte encapsulation process (9).

The high expression level of BgTEP transcripts in snail hemocytes, the specialized circulating immune cells in *Biomphalaria* snails, correlates with results obtained for *A. gambiae* (8), thereby emphasizing its potential immune function. However, high expression levels were also observed in other tissues including ovotestis. Ovotestis is the center of production for eggs and spermatozoids and is of paramount importance for putative immune molecule transmission to progeny. In a previous study, *B. glabrata* was shown to invest in its offspring’s protection (60), and several immune factors including BgTEP (called A2M when published) were recovered by proteomic analysis in egg masses (61). Production of BgTEP transcripts in ovotestis is thus highly relevant with this potential transfer of protection to eggs and progeny. Moreover, a recent study showed in *Anopheles* that during spermatogenesis, AgTEP1 binds to and removes damaged cells, increasing fertility rates (62).

In naïve snails, BgTEP is constitutively secreted in the hemolymph, and expressed at high levels in circulating immune cells. Western blot on plasma, using the anti-BgTEP-PEP antibody, revealed the presence of full length, as well as several processed forms, of BgTEP. This suggests that BgTEP undergoes a cleavage by an undefined plasmatic factor, similar in the case of AgTEP1 (8, 63). In *A. gambiae*, the AgTEP1 is found in full-length and in a processed form called TEP-cut, which allows for a complex pattern of TEP that is ready to respond to a pathogen attack (8). In hemolymph, the AgTEP1 is maintained by a complex of two proteins, such as APL1 and LRIM1, to stabilize the processed form and to avoid the unspecific binding of the thioester domain to non-relevant substrates (8, 63). In vertebrates, the complement component pathway displays a major role in the innate immune system. The complement component C3 activation is finely regulated by a series of proteolytic cleavages leading to the formation of different fragments of C3 such as C3a, C3b, iC3b, and C3dg. Some proteolytic fragments, such as the small complement fragment C3a, mediate chemotaxis and local inflammation. C3b acts as an opsonin by enhancing cellular phagocytosis by binding to the pathogen’s surface. The C3b-derived fragment, iC3b, and C3dg can bind to the pathogen and promote its uptake (64–66).

Interestingly, an immune labeling on hemocytes revealed that only a subset of blast-like cells is positive for BgTEP (Figure 7A). This observation possibly suggests that more hemocyte subtypes or a differential maturation exist in the *B. glabrata* hemolymph when compared with the ones previously estimated solely on the basis of cell morphology analysis. Further investigations are needed to confirm this observation through a characterization of functions for each hemocyte type.

Through a targeted interactome approach, we observed that BgTEP is retrieved bound to all intruders tested in this study, in its full length and in processed forms (Figure 6). This suggests that the full length of BgTEP bound on the intruders surface can be cleaved by a proteolytic cascade or that processed forms can bind directly. These results are consistent with the previously observed binding of AgTEP1 to bacteria that was shown to occur in both thioester-dependent and thioester-independent scenarios (8). This could also indicate that BgTEP is probably able to bind intruder surfaces directly or indirectly associated with other immune relevant partners. As for mosquito TEP1 (8), no apparent electrophoretic shift was observed between bands obtained from naïve and interactome conditions. But, we cannot exclude that some bands might also correspond to proteolytic fragment of BgTEP covalently associated with intruder proteins as shown for a recombinant LvTEP1 from a crustacean, *L. vannamei* (14).

After 3 h of interaction, fewer full length and 30 kDa processed forms of BgTEP were recovered in forms bound to the surface of *E. coli, S. cerevisiae*, and *S. mansoni*, indicating a time-dependent processing compared with a shorter treatment (Figure 6). New forms of BgTEP appeared after 3 h of incubation with yeast and bacteria, which could result from the processing of already bound full-length protein (Figure 6A). Another striking result is the difference observed for the binding of BgTEP between miracidia and sporocysts, which are two successive developmental stages of *S. mansoni* parasite (Figure 6B). Interestingly, more processed forms of BgTEP were recovered bound to sporocysts than to miracidia, and no bands were detected with miracidia after 3 h, suggesting a higher specificity of BgTEP for sporocyst stage than for miracidia. Such a result is not surprising as a sporocyst is the result of miracidia transformation which consists mainly of the loss of epidermal ciliated plates and tegument renewal that occurs in the early hours after infection. Several proteomic and glycomic studies have highlighted differences from one developmental stage to another (67, 68). These results would suggest a subtle ability of the snail immune machinery to distinguish between various developmental stages of the parasite. Moreover, we showed that methylamine treatment inhibits the binding of either full length or larger processed forms of BgTEP to Sp1 surface (Figure S3 in Supplementary Material). Such result clearly indicates that the binding of BgTEP to Sp1 surface is mediated by its thioester site.

Collectively, these results clearly indicate that BgTEP can be associated with the surface of live intruders and could be differentially processed depending on the intruder type. For the first time, we also approached the dynamic of immune complex formation with a selective processing of bound TEP between intruders. So even if intruders were sensed by this complement-like factor (51), other maturation factors may be involved to induce an appropriate immune response. Nevertheless, the binding mechanisms are still unclear and need to be deeply characterized.

As BgTEP can interact with several intruders, we investigated the relative expression of BgTEP transcript by quantitative RT-PCR following various immune challenges (Figure 5). BgTEP expression is modulated, regardless of the nature of the intruders used for the stimulation step. *E. coli* and *S. cerevisiae* challenges decreased its expression, while *M. luteus* and *S. mansoni* challenges upregulated it, as previously detailed in the transcriptomic analysis of the snail immune response after bacterial and fungal infections (53). Interestingly, *S. mansoni* is the only one that induced a constant increase from 6 to 24 h of BgTEP transcript expression suggesting a role of first importance in the interaction between *S. mansoni* and *B. glabrata*, which supports the first identification of BgTEP in a host–parasite immune complex (42). Although we observed many BgTEP-positive hemocytes converging toward the encapsulated parasite and surrounding the hemocyte capsule (Figure S4 in Supplementary Material), a typical associated immune response with an incompatible strain of *S. mansoni*. This observation suggests the participation of BgTEP-positive hemocytes in the recruitment of capsule-forming hemocytes on the site of infection, potentially *via* a putative α-2-macroglobulin receptor on their membrane (41, 69, 70). Furthermore, hemocytes converging to the site of infection may also support a potential chemotaxis property of BgTEP due to a cleavage of bound TEP into small ANA-like fragments.

This study provides new insights about the potential immune function of BgTEP. We demonstrate that its constitutive production by hemocytes must be modulated by immune challenges, and that the full protein and its proteolytic fragments are able to bind the surface of different intruders before and after specific cleavage maturing processes. Even though the precise binding mechanism needs further characterization, our results suggest that BgTEP displayed an immune role by targeting intruder surface.

In this work, we report the first characterization of an iTEP displaying a dual-role, whose existence was previously argued (1). As described before, BgTEP acts as an antiprotease (43), but in this study we demonstrate that BgTEP can also bind to different intruders, including the *S. mansoni* parasite, and could participate in their elimination.

In conclusion, a more precise functional characterization is necessary to decipher the key role of the BgTEP and its action dynamics during the immune response of the snail. To that end, a loss of gene function by CRISPR/cas9 technology or RNAi would be considered. Also, the nature and function of proteolytic products of BgTEP remain unknown and must be explored to elucidate host–pathogen interaction. Indeed, some pathogens circumvent the host immune response by blocking or miscleaving complement components (71–73).

## Ethics Statement

Our laboratory holds permit # A66040 for experiments on animals, which was obtained from the French Ministry of Agriculture and Fisheries and the French Ministry of National Education, Research, and Technology. The housing, breeding, and care of the utilized animals followed the ethical requirements of our country. The experimenter possesses an official certificate for animal experimentation from both of the above-listed French ministries (Decree # 87–848, October 19, 1987). The various protocols used in this study have been approved by the French veterinary agency of the DRAAF Languedoc-Roussillon (Direction Régionale de l’Alimentation, de l’Agriculture et de la Forêt), Montpellier, France (authorization # 007083).

## Author Contributions

AP performed interactome and immunoblotting, as well as quantitative RT-PCR experiments, FACS and microscopy analysis. RG designed the research, performed the phylogenetic analysis, and participated in Western blot experiments. SP performed interactome experiments. JP performed microscopy analysis. FN performed immunohistological experiment. BG substantially participated in conception and improvement of research. DD designed the research and performed genomic organization analysis of BgTEP gene. All the authors participated to manuscript writing, editing, critical reviewing, and they all approved the final draft.

## Conflict of Interest Statement

The authors declare that the research was conducted in the absence of any commercial or financial relationships that could be construed as a potential conflict of interest.

## Supplementary Material

Figure S1 Web-logo representation of the consensus sequence of intron–exon junction in BgTEP gene.

Figure S2 May–Grünwald–Giemsa staining of *Biomphalaria glabrata* hemocytes revealing the three major hemocyte subtypes: hyalinocytes, granulocytes, and blast-like cells.

Figure S3 Methylamine treatment effect on BgTEP binding to Sp1. 500 Sp1 were incubated 30 min at 26°C with either (1) 1 mL of Chernin’s balanced salt solution (CBSS) buffer, (2) 1 mL of cell-free plasma, or (3) 1 mL of cell-free plasma + methylamine (2 mM). Sp1 were further centrifuged and washed with CBSS buffer. Proteins from Sp1 pellet were subsequently extracted with Laemmli buffer and run on a 7.5% SDS-PAGE, before being transferred onto a 0.2 µm PVDF membrane. Western blot against BgTEP was performed as in Figure 6, using anti-BgTEP-RP antibody.

Figure S4 Histological section (10 µm thickness) of *Biomphalaria glabrata* paraffin embedded tissue in which can be observed the encapsulation of a *Schistosoma mansoni* parasite by hemocytes 24 h post-infection. White arrows show the BgTEP-positive cells (red) around and in the capsule. The BgTEP protein is detected by immunolocalization using anti BgTEP-PEP antibody.

## References

[1] Williams M Baxter R The structure and function of thioester-containing proteins in arthropods. Biophys Rev (2014) 6:261–72. 10.1007/s12551-014-0142-6 PMC5376097 28510031

[2] Shokal U Eleftherianos I. Evolution and function of thioester-containing proteins and the complement system in the innate immune response. Front Immunol (2017) 8:759. 10.3389/fimmu.2017.00759 28706521 PMC5489563

[3] Dodds MW Law ASK The phylogeny and evolution of the thioester bond-containing proteins C3, C4 and α2-macroglobulin. Immunol Rev (1998) 166:15–26. 10.1111/j.1600-065X.1998.tb01249.x 9914899

[4] Lagueux M Perrodou E Levashina EA Capovilla M Hoffmann JA. Constitutive expression of a complement-like protein in toll and JAK gain-of-function mutants of *Drosophila*. Proc Natl Acad Sci U S A (2000) 97:11427–32. 10.1073/pnas.97.21.11427 11027343 PMC17216

[5] Stroschein-Stevenson SL Foley E O’Farrell PH Johnson AD. Identification of *Drosophila* gene products required for phagocytosis of *Candida albicans*. PLoS Biol (2006) 4:e4. 10.1371/journal.pbio.0040004 16336044 PMC1310651

[6] de Almeida Oliveira G Lieberman J Barillas-Mury C. Epithelial nitration by a peroxidase/NOX5 system mediates mosquito antiplasmodial immunity. Science (2012) 335:856–9. 10.1126/science.1209678 22282475 PMC3444286

[7] Baxter RHG Contet A Krueger K. Arthropod innate immune systems and vector-borne diseases. Biochemistry (2017) 56:907–18. 10.1021/acs.biochem.6b00870 28072517 PMC5741185

[8] Levashina EA Moita LF Blandin S Vriend G Lagueux M Kafatos FC. Conserved role of a complement-like protein in phagocytosis revealed by dsRNA knockout in cultured cells of the mosquito, *Anopheles gambiae*. Cell (2001) 104:709–18. 10.1016/S0092-8674(01)00267-7 11257225

[9] Blandin S Levashina EA. Thioester-containing proteins and insect immunity. Mol Immunol (2004) 40:903–8. 10.1016/j.molimm.2003.10.010 14698229

[10] Buresova V Hajdusek O Franta Z Loosova G Grunclova L Levashina EA Functional genomics of tick thioester-containing proteins reveal the ancient origin of the complement system. J Innate Immun (2011) 3:623–30. 10.1159/000328851 21811049

[11] Sekiguchi R Fujito NT Nonaka M. Evolution of the thioester-containing proteins (TEPs) of the arthropoda, revealed by molecular cloning of TEP genes from a spider, *Hasarius adansoni*. Dev Comp Immunol (2012) 36:483–9. 10.1016/j.dci.2011.05.003 21663759

[12] Urbanová V Šíma R Šauman I Hajdušek O Kopáček P. Thioester-containing proteins of the tick *Ixodes ricinus*: gene expression, response to microbial challenge and their role in phagocytosis of the yeast *Candida albicans*. Dev Comp Immunol (2015) 48:55–64. 10.1016/j.dci.2014.09.004 25224405

[13] Wu C Noonin C Jiravanichpaisal P Söderhäll I Söderhäll K. An insect TEP in a crustacean is specific for cuticular tissues and involved in intestinal defense. Insect Biochem Mol Biol (2012) 42:71–80. 10.1016/j.ibmb.2011.10.006 22193393

[14] Li C Li H Xiao B Chen Y Wang S Lu K Identification and functional analysis of a TEP gene from a crustacean reveals its transcriptional regulation mediated by NF-κB and JNK pathways and its broad protective roles against multiple pathogens. Dev Comp Immunol (2017) 70:45–58. 10.1016/j.dci.2017.01.005 28069434

[15] Fujito NT Sugimoto S Nonaka M Evolution of thioester-containing proteins revealed by cloning and characterization of their genes from a cnidarian sea anemone, *Haliplanella lineate*. Dev Comp Immunol (2010) 34:775–84. 10.1016/j.dci.2010.02.011 20188753

[16] Zhang H Song L Li C Zhao J Wang H Gao Q Molecular cloning and characterization of a thioester-containing protein from Zhikong scallop *Chlamys farreri*. Mol Immunol (2007) 44:3492–500. 10.1016/j.molimm.2007.03.008 17498803

[17] Yazzie N Salazar KA Castillo MG. Identification, molecular characterization, and gene expression analysis of a CD109 molecule in the *Hawaiian bobtail* squid *Euprymna scolopes*. Fish Shellfish Immunol (2015) 44:342–55. 10.1016/j.fsi.2015.02.036 25742727

[18] Vierstraete E Verleyen P Baggerman G D’Hertog W Van Den Bergh G Arckens L A proteomic approach for the analysis of instantly released wound and immune proteins in *Drosophila melanogaster* hemolymph. Proc Natl Acad Sci U S A (2004) 101:470–5. 10.1073/pnas.0304567101 14707262 PMC327171

[19] Blandin SA Marois E Levashina EA. Antimalarial responses in *Anopheles gambiae*: from a complement-like protein to a complement-like pathway. Cell Host Microbe (2008) 3:364–74. 10.1016/j.chom.2008.05.007 18541213

[20] Baxter RHG Chang C-I Chelliah Y Blandin S Levashina EA Deisenhofer J. Structural basis for conserved complement factor-like function in the antimalarial protein TEP1. Proc Natl Acad Sci U S A (2007) 104:11615–20. 10.1073/pnas.0704967104 17606907 PMC1905922

[21] Yassine H Osta MA. *Anopheles gambiae* innate immunity. Cell Microbiol (2010) 12:1–9. 10.1111/j.1462-5822.2009.01388.x 19804484

[22] Yassine H Kamareddine L Osta MA. The mosquito melanization response is implicated in defense against the entomopathogenic fungus *Beauveria bassiana*. PLoS Pathog (2012) 8:e1003029. 10.1371/journal.ppat.1003029 23166497 PMC3499577

[23] Smith RC Barillas-Mury C Jacobs-Lorena M. Hemocyte differentiation mediates the mosquito late-phase immune response against *Plasmodium* in *Anopheles gambiae*. Proc Natl Acad Sci U S A (2015) 112:E3412–20. 10.1073/pnas.1420078112 26080400 PMC4491748

[24] Wertheim B Kraaijeveld AR Schuster E Blanc E Hopkins M Pletcher SD Genome-wide gene expression in response to parasitoid attack in *Drosophila*. Genome Biol (2005) 6:R94. 10.1186/gb-2005-6-11-r94 16277749 PMC1297650

[25] Bou Aoun R Hetru C Troxler L Doucet D Ferrandon D Matt N Analysis of thioester-containing proteins during the innate immune response of *Drosophila melanogaster*. J Innate Immun (2010) 3:52–64. 10.1159/000321554 21063077 PMC3031515

[26] Arefin B Kucerova L Dobes P Markus R Strnad H Wang Z Genome-wide transcriptional analysis of *Drosophila* larvae infected by entomopathogenic nematodes shows involvement of complement, recognition and extracellular matrix proteins. J Innate Immun (2014) 6:192–204. 10.1159/000353734 23988573 PMC6741570

[27] Dostálová A Rommelaere S Poidevin M Lemaitre B. Thioester-containing proteins regulate the toll pathway and play a role in *Drosophila* defence against microbial pathogens and parasitoid wasps. BMC Biol (2017) 15:79. 10.1186/s12915-017-0408-0 28874153 PMC5584532

[28] Lin L Rodrigues FSLM Kary C Contet A Logan M Baxter RHG Complement-related regulates autophagy in neighboring cells. Cell (2017) 170(1):158–71. 10.1016/j.cell.2017.06.018 28666117 PMC5533186

[29] Batz T Forster D Luschnig S. The transmembrane protein macroglobulin complement-related is essential for septate junction formation and epithelial barrier function in *Drosophila*. Development (2014) 141(4):899–908. 10.1242/dev.102160 24496626

[30] Hall S Bone C Oshima K Zhang L McGraw M Lucas B Macroglobulin complement-related encodes a protein required for septate junction organization and paracellular barrier function in *Drosophila*. Development (2014) 141(4):889–98. 10.1242/dev.102152 24496625 PMC3912832

[31] Xue Z Wang L Liu Z Wang W Liu C Song X The fragmentation mechanism and immune-protective effect of CfTEP in the scallop *Chlamys farreri*. Dev Comp Immunol (2017) 76:220–8. 10.1016/j.dci.2017.06.005 28625746

[32] King CH Dickman K Tisch DJ. Reassessment of the cost of chronic helmintic infection: a meta-analysis of disability-related outcomes in endemic schistosomiasis. Lancet (2005) 365:1561–9. 10.1016/S0140-6736(05)66457-4 15866310

[33] Mitta G Adema CM Gourbal B Loker ES Theron A. Compatibility polymorphism in snail/schistosome interactions: from field to theory to molecular mechanisms. Dev Comp Immunol (2012) 37:1–8. 10.1016/j.dci.2011.09.002 21945832 PMC3645982

[34] Portet A Pinaud S Tetreau G Galinier R Cosseau C Duval D Integrated multi-omic analyses in *Biomphalaria*-*Schistosoma* dialogue reveal the immunobiological significance of FREP-SmPoMuc interaction. Dev Comp Immunol (2017) 75:16–27. 10.1016/j.dci.2017.02.025 28257854

[35] Tennessen JA Théron A Marine M Yeh J-Y Rognon A Blouin MS. Hyperdiverse gene cluster in snail host conveys resistance to human schistosome parasites. PLoS Genet (2015) 11:e1005067. 10.1371/journal.pgen.1005067 25775214 PMC4361660

[36] Roger E Gourbal B Grunau C Pierce RJ Galinier R Mitta G Expression analysis of highly polymorphic mucin proteins (Sm PoMuc) from the parasite *Schistosoma mansoni*. Mol Biochem Parasitol (2008) 157:217–27. 10.1016/j.molbiopara.2007.11.015 18187213

[37] Roger E Grunau C Pierce RJ Hirai H Gourbal B Galinier R Controlled chaos of polymorphic mucins in a metazoan parasite (*Schistosoma mansoni*) interacting with its invertebrate host (*Biomphalaria glabrata*). PLoS Negl Trop Dis (2008) 2:e330 10.1371/journal.pntd.0000330 19002242 PMC2576457

[38] Roger E Mitta G Moné Y Bouchut A Rognon A Grunau C Molecular determinants of compatibility polymorphism in the *Biomphalaria glabrata*/*Schistosoma mansoni* model: new candidates identified by a global comparative proteomics approach. Mol Biochem Parasitol (2008) 157:205–16. 10.1016/j.molbiopara.2007.11.003 18083248

[39] Adema CM Hertel LA Miller RD Loker ES. A family of fibrinogen-related proteins that precipitates parasite-derived molecules is produced by an invertebrate after infection. Proc Natl Acad Sci U S A (1997) 94:8691–6. 10.1073/pnas.94.16.8691 9238039 PMC23082

[40] Zhang S-M Adema CM Kepler TB Loker ES. Diversification of Ig superfamily genes in an invertebrate. Science (2004) 305:251–4. 10.1126/science.1088069 15247481

[41] Pila EA Li H Hambrook JR Wu X Hanington PC Schistosomiasis from a snail’s perspective: advances in snail immunity. Trends Parasitol (2017) 11:845–57. 10.1016/j.pt.2017.07.006 28803793

[42] Moné Y Gourbal B Duval D Du Pasquier L Kieffer-Jaquinod S Mitta G. A large repertoire of parasite epitopes matched by a large repertoire of host immune receptors in an invertebrate host/parasite model. PLoS Negl Trop Dis (2010) 4:e813. 10.1371/journal.pntd.0000813 20838648 PMC2935394

[43] Bender RC Bayne CJ Purification and characterization of a tetrameric alpha-macroglobulin proteinase inhibitor from the gastropod mollusc *Biomphalaria glabrata*. Biochem J (1996) 316:893–900. 10.1042/bj3160893 8670168 PMC1217434

[44] Galinier R Roger E Mone Y Duval D Portet A Pinaud S A multistrain approach to studying the mechanisms underlying compatibility in the interaction between *Biomphalaria glabrata* and *Schistosoma mansoni*. PLoS Negl Trop Dis (2017) 11(3):e0005398. 10.1371/journal.pntd.0005398 28253264 PMC5349689

[45] Dheilly NM Duval D Mouahid G Emans R Allienne J-F Galinier R A family of variable immunoglobulin and lectin domain containing molecules in the snail *Biomphalaria glabrata*. Dev Comp Immunol (2015) 48:234–43. 10.1016/j.dci.2014.10.009 25451302 PMC4255472

[46] Zhang Y Skolnick J. TM-align: a protein structure alignment algorithm based on the TM-score. Nucleic Acids Res (2005) 33:2302–9. 10.1093/nar/gki524 15849316 PMC1084323

[47] Zhang Y. I-TASSER server for protein 3D structure prediction. BMC Bioinformatics (2008) 9:40. 10.1186/1471-2105-9-40 18215316 PMC2245901

[48] Roy A Kucukural A Zhang Y. I-TASSER: a unified platform for automated protein structure and function prediction. Nat Protoc (2010) 5:725–38. 10.1038/nprot.2010.5 20360767 PMC2849174

[49] Tamura K Peterson D Peterson N Stecher G Nei M Kumar S. MEGA5: molecular evolutionary genetics analysis using maximum likelihood, evolutionary distance, and maximum parsimony methods. Mol Biol Evol (2011) 28:2731–9. 10.1093/molbev/msr121 21546353 PMC3203626

[50] Sminia T Barendsen L A comparative morphological and enzyme histochemical study on blood cells of the freshwater snails *Lymnaea stagnalis, Biomphalaria glabrata*, and *Bulinus truncatus*. J Morphol (1980) 165:31–9. 10.1002/jmor.1051650104 30153712

[51] Tetreau G Pinaud S Portet A Galinier R Gourbal B Duval D. Specific pathogen recognition by multiple innate immune sensors in an invertebrate. Front Immunol (2017) 8:1249. 10.3389/fimmu.2017.01249 29051762 PMC5633686

[52] Galinier R Portela J Moné Y Allienne JF Henri H Delbecq S Biomphalysin, a new β pore-forming toxin involved in *Biomphalaria glabrata* immune defense against *Schistosoma mansoni*. PLoS Pathog (2013) 9:e1003216. 10.1371/journal.ppat.1003216 23555242 PMC3605176

[53] Deleury E Dubreuil G Elangovan N Wajnberg E Reichhart JM Gourbal B Specific versus non-specific immune responses in an invertebrate species evidenced by a comparative de novo sequencing study. PLoS One (2012) 7:e32512. 10.1371/journal.pone.0032512 22427848 PMC3299671

[54] Adema CM Hillier LW Jones CS Loker ES Knight M Minx P Whole genome analysis of a schistosomiasis-transmitting freshwater snail. Nat Commun (2017) 8:15451. 10.1038/ncomms15451 28508897 PMC5440852

[55] Solomon KR Sharma P Chan M Morrison PT Finberg RW CD109 represents a novel branch of the α2-macroglobulin/complement gene family. Gene (2004) 327(2):171–83. 10.1016/j.gene.2003.11.025 14980714

[56] Bender RC Fryer SE Bayne CJ. Proteinase inhibitory activity in the plasma of a mollusc: evidence for the presence of alpha-macroglobulin in *Biomphalaria glabrata*. Comp Biochem Physiol (1992) 102:821–4. 1382916 10.1016/0305-0491(92)90086-7

[57] Fryer SE Bender RC Bayne CJ. Inhibition of cysteine proteinase from *Schistosoma mansoni* larvae by alpha-macroglobulin from the plasma of *Biomphalaria glabrata*. J Parasitol (1996) 82:343–7. 10.2307/3284177 8604113

[58] Prosper JYA Characterization of CD109. Toronto: University of Toronto (2011).

[59] Zhang H Wang L Song L Zhao J Qiu L Gao Y The genomic structure, alternative splicing and immune response of *Chlamys farreri* thioester-containing protein. Dev Comp Immunol (2009) 33:1070–6. 10.1016/j.dci.2009.05.007 19467260

[60] Baron OL Van West P Industri B Ponchet M Dubreuil G Gourbal B Parental transfer of the antimicrobial protein LBP/BPI protects *Biomphalaria glabrata* eggs against oomycete infections. PLoS Pathog (2013) 9:e1003792. 10.1371/journal.ppat.1003792 24367257 PMC3868517

[61] Hathaway JJM Adema CM Stout BA Mobarak CD Loker ES. Identification of protein components of egg masses indicates parental investment in immunoprotection of offspring by *Biomphalaria glabrata* (Gastropoda, Mollusca). Dev Comp Immunol (2010) 34:425–35. 10.1016/j.dci.2009.12.001 19995576 PMC2813990

[62] Pompon J Levashina EA. A new role of the mosquito complement-like cascade in male fertility in *Anopheles gambiae*. PLoS Biol (2015) 13:e1002255. 10.1371/journal.pbio.1002255 26394016 PMC4579081

[63] Shokal U Kopydlowski H Eleftherianos I. The distinct function of Tep2 and Tep6 in the immune defense of *Drosophila melanogaster* against the pathogen *Photorhabdus*. Virulence (2017) 5594:1–15. 10.1080/21505594.2017.1330240 28498729 PMC5810505

[64] Hamad OA Nilsson PH Wouters D Lambris JD EkDahl KN Nilsson B. Complement component C3 binds to activated normal platelets without preceding proteolytic activation and promotes binding to complement receptor 1. J Immunol (2010) 184:2686–92. 10.4049/jimmunol.0902810 20139276 PMC2953618

[65] Feng S Liang X Kroll MH Chung DW Afshar-Kharghan V. von Willebrand factor is a cofactor in complement regulation. Blood (2015) 125:1034–7. 10.1182/blood-2014-06-585430 25395424 PMC4319234

[66] Foley JH Peterson EA Lei V Wan LW Krisinger MJ Conway EM. Interplay between fibrinolysis and complement: plasmin cleavage of iC3b modulates immune responses. J Thromb Haemost (2015) 13:610–8. 10.1111/jth.12837 25556624

[67] Hokke CH Deelder AM Hoffmann KF Wuhrer M Glycomics-driven discoveries in schistosome research. Exp Parasitol (2007) 117(3):275–83. 10.1016/j.exppara.2007.06.003 17659278

[68] Peterson NA Hokke CH Deelder AM Yoshino TP Glycotope analysis in miracidia and primary sporocysts of Schistosoma mansoni: differential expression during the miracidium-to-sporocyst transformation. Int J Parasitol (2009) 39(12):1331–44. 10.1016/j.ijpara.2009.06.002 19545571 PMC3740939

[69] Coustau C Gourbal B Duval D Yoshino TP Adema CM Mitta G. Advances in gastropod immunity from the study of the interaction between the snail *Biomphalaria glabrata* and its parasites: a review of research progress over the last decade. Fish Shellfish Immunol (2015) 46:5–16. 10.1016/j.fsi.2015.01.036 25662712

[70] Paul LM Carlin ER Jenkins MM Tan AL Barcellona CM Nicholson CO Dengue virus antibodies enhance Zika virus infection. Clin Transl Immunol (2016) 5:e117 10.1038/cti.2016.72 PMC5192063 28090318

[71] Jusko M Potempa J Kantyka T Bielecka E Miller HK Kalinska M Staphylococcal proteases aid in evasion of the human complement system. J Innate Immun (2014) 6:31–46. 10.1159/000351458 23838186 PMC3972074

[72] Johnson JB Borisevich V Rockx B Parks GD. A novel factor I activity in Nipah virus inhibits human complement pathways through cleavage of C3b. J Virol (2015) 89:989–98. 10.1128/JVI.02427-14 25355897 PMC4300667

[73] Luo S Dasari P Reiher N Hartmann A Jacksch S Wende E The secreted *Candida albicans* protein Pra1 disrupts host defense by broadly targeting and blocking complement C3 and C3 activation fragments. Mol Immunol (2018) 93:266–77. 10.1016/j.molimm.2017.07.010 28860090

