# Supplementary figures and images for: BgTEP: An Antiprotease Involved in Innate Immune Sensing in Biomphalaria glabrata
Source: Front Immunol. 2018 May 29;9:1206. doi: 10.3389/fimmu.2018.01206 (PMC5989330; doi:10.3389/fimmu.2018.01206)

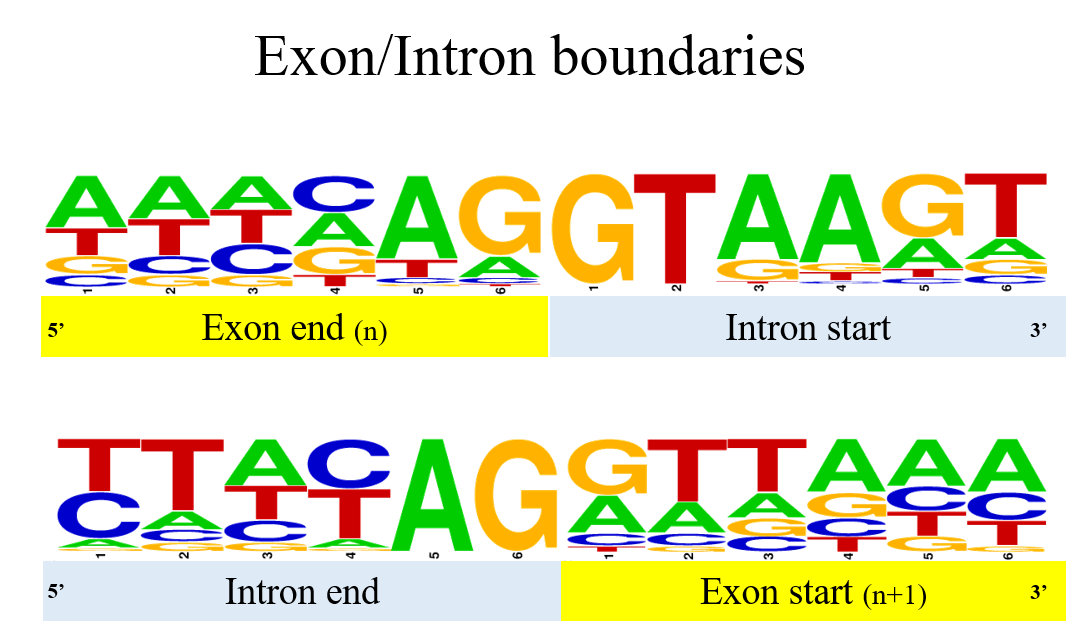

Supplement: Figure S1 — Web-logo representation of the consensus sequence of intron–exon junction in BgTEP gene. [file image_1.tif]

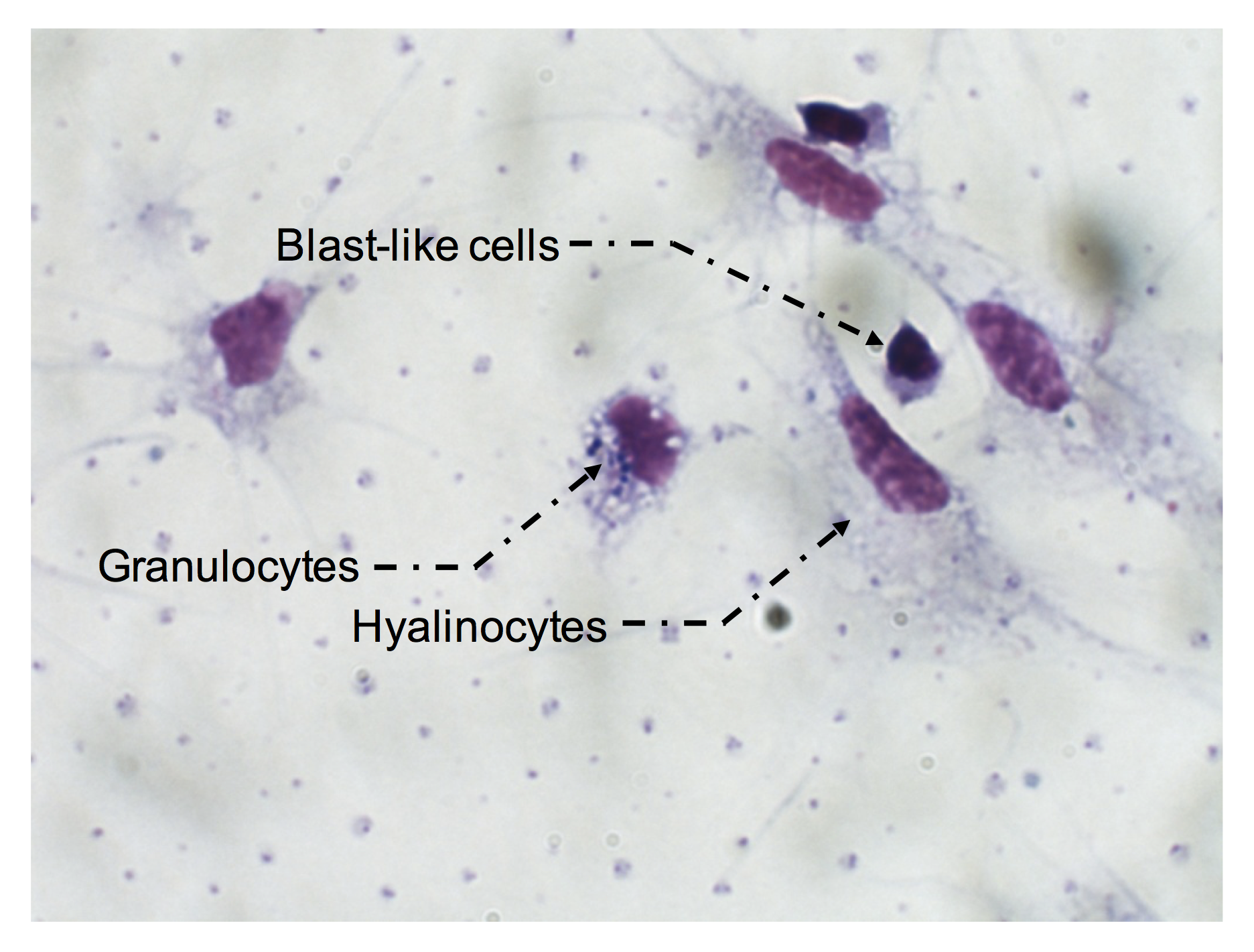

Supplement: Figure S2 — May–Grünwald–Giemsa staining of Biomphalaria glabrata hemocytes revealing the three major hemocyte subtypes: hyalinocytes, granulocytes, and blast-like cells. [file image_2.tiff]

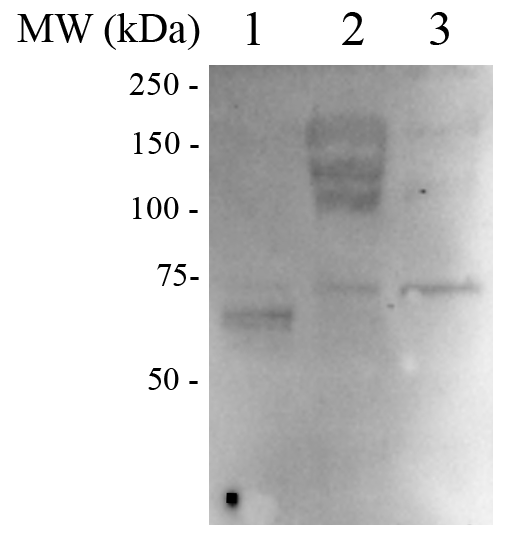

Supplement: Figure S3 — Methylamine treatment effect on BgTEP binding to Sp1. 500 Sp1 were incubated 30 min at 26°C with either (1) 1 mL of Chernin’s balanced salt solution (CBSS) buffer, (2) 1 mL of cell-free plasma, or (3) 1 mL of cell-free plasma + methylamine (2 mM). Sp1 were further centrifuged and washed with CBSS buffer. Proteins from Sp1 pellet were subsequently extracted with Laemmli buffer and run on a 7.5% SDS-PAGE, before being transferred onto a 0.2 µm PVDF membrane. Western blot against BgTEP was performed as in Figure 6, using anti-BgTEP-RP antibody. [file image_3.tiff]

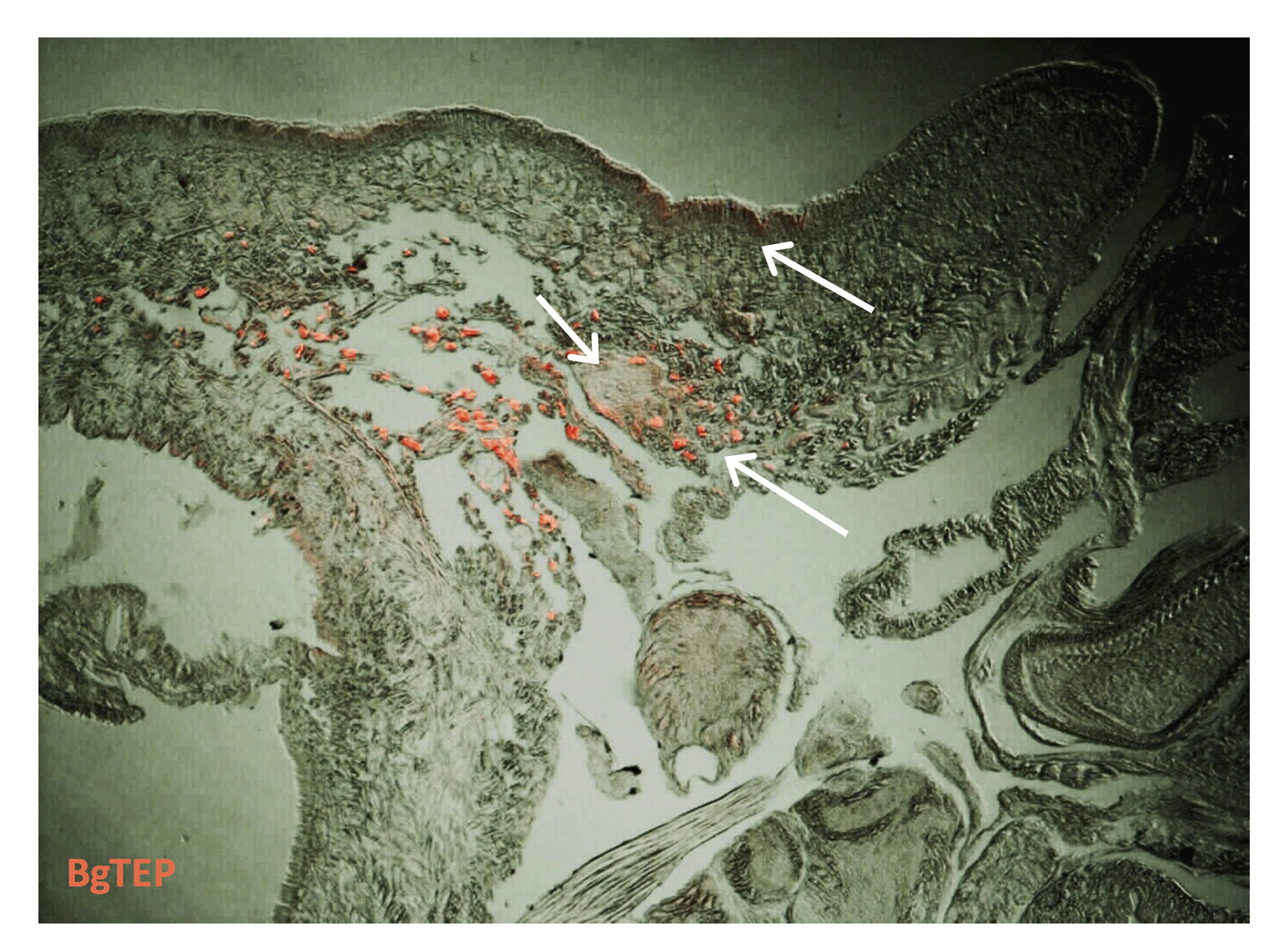

Supplement: Figure S4 — Histological section (10 µm thickness) of Biomphalaria glabrata paraffin embedded tissue in which can be observed the encapsulation of a Schistosoma mansoni parasite by hemocytes 24 h post-infection. White arrows show the BgTEP-positive cells (red) around and in the capsule. The BgTEP protein is detected by immunolocalization using anti BgTEP-PEP antibody. [file image_4.tiff]
